# Sarcopenia and Insulin Resistance Collective Effect on Atrial Fibrillation Risk: A Non‐Diabetic Elderly Cohort Study

**DOI:** 10.1002/jcsm.13736

**Published:** 2025-02-17

**Authors:** Weike Liu, Xin Wang, Yuqi Guo, Yumei Gao, Huajing Song, Yanli Yao, Hua Zhang, Zhendong Liu, Juan Wang

**Affiliations:** ^1^ Department of Cardiology The Second Affiliated Hospital of Harbin Medical University Harbin Heilongjiang China; ^2^ Department of Cardiology The Second Hospital of Shandong University Jinan Shandong China; ^3^ Department of Cardiology The First Affiliated Hospital of Shandong First Medical University Jinan Shandong China; ^4^ Cardio‐Cerebrovascular Control and Research Center, Clinical and Basic Medicine College Shandong First Medical University & Shandong Academy of Medical Sciences Jinan Shandong China; ^5^ Department of Cardiology Hekou District People Hospital Dongying Shandong China

**Keywords:** atrial fibrillation, dual‐trajectory analysis, insulin resistance, muscle mass loss, non‐diabetes

## Abstract

**Background:**

Appendicular skeletal muscle mass index (ASMI), a crucial indicator of sarcopenia and estimated glucose disposal rate (eGDR), a surrogate marker of insulin resistance (IR), are associated with the risk of cardiovascular diseases. However, it remains unclear whether the collective effects, including the impact of the temporal progression of ASMI and eGDR, affect atrial fibrillation (AF) risk. This study aims to elucidate the association between the collective effects of ASMI and eGDR and AF risk in the non‐diabetic older population.

**Methods:**

A total of 8060 non‐diabetic older individuals from a community‐based cohort study were used to prospectively analyse the association between the collective effects of baseline ASMI and eGDR and AF risk. Among them, 7651 were eligible and used for dual‐trajectory analysis of the association between dual trajectory of ASMI and eGDR and AF risk. The temporal development of ASMI and eGDR over time was determined using a dual‐trajectory model. Statistical analyses involved restricted cubic splines and Fine–Gray competing risk models, adjusting for potential confounders.

**Results:**

In the prospective analysis, the hazard ratio (HR) of AF was 1.762 (95% confidence interval [CI]: 1.528–2.032) in the low ASMI group compared to the normal ASMI group in total participants. Restricted cubic splines analysis demonstrated L‐shaped associations between AF risk and ASMI and eGDR, with inflection points at 7.23 kg/m^2^ and 7.85 mg/kg/min, respectively. Low ASMI and moderate and low eGDR exhibited a significant interplay for increasing AF risk (HR: 1.290 and 1.666, 95% CI: 1.136–1.464 and 1.492–1.861, respectively, *p*_adj._ < 0.001). One‐SD increment ASMI and eGDR synergistically reduced AF risk (HR: 0.896, 95% CI: 0.839–0.957, *p*_adj._ < 0.001). In the dual‐trajectory analysis for total participants, five distinct dual trajectories of ASMI and eGDR were identified. Group 4, characterized by moderate‐stable ASMI and moderate‐stable eGDR, exhibited the lowest incidence of AF (7.03 per 1000 person‐years) and was used as a reference for further analyses. Group 1, characterized by high‐decrease ASMI and high‐decrease eGDR, had the highest AF risk (HR: 2.255, 95% CI: 1.769–2.876, *p*_adj._ < 0.001), followed by Group 5, with high‐decrease ASMI and low‐stable eGDR (HR: 1.893, 95% CI: 1.491–2.403, *p*_adj._ < 0.001) when compared to Group 4 after adjustment for potential confounders including baseline ASMI and eGDR.

**Conclusions:**

The collective effects of ASMI and eGDR are significantly associated with AF risk in the non‐diabetic older population. Collective management of skeletal muscle mass and IR might be a useful and effective management strategy for preventing and controlling AF.

**Abbreviations**
AF: atrial fibrillation
AIC: Akaike's information criterion
APP: average posterior probability
ASMI: appendicular skeletal muscle mass index
BIC: Bayesian information criterion
CCA‐IMT: common carotid artery intima–media thickness
COPD: chronic obstructive pulmonary disease
DBP: diastolic blood pressure
eGDR: estimated glucose disposal rate
eGFR: estimated glomerular filtration rate
FPG: fasting plasma glucose
HbA1c: haemoglobin A1c
IR: insulin resistance
SBP: systolic blood pressure
TCHO: lipid profile data including total cholesterol
WC: waist circumference

## 1 Introduction

Atrial fibrillation (AF) is a common and life‐threatening cardiac arrhythmia, increasing the risk of sudden cardiac death, stroke, heart failure and cognitive impairment or dementia [1, 2, 3, 4, 5], and there are approximately 60 million individuals affected by AF worldwide [1, 2, 3, 4, 5]. The prevalence of AF increases with age and is estimated to double every decade beginning at 60 years of age [1, 2, 3, 4, 5] and poses heavy social and personal healthcare burdens and has become an urgent public health challenge [1, 2, 3, 4, 5]. As the global population ages, identifying risk factors is crucial for coping with the challenges caused by AF.

Sarcopenia is a geriatric syndrome characterized by a progressive and age‐related loss of skeletal muscle mass and strength and/or reduced physical performance [6, 7]. It has been demonstrated that sarcopenia is closely associated with the heightened risks of physical disability, fractures, dementia, deficient quality of life and mortality [3, 7, 8, 9]. Recently, there has been a burgeoning interest in understanding the role of sarcopenia in cardiovascular diseases [3, 9, 10, 11]. However, there is currently limited studies on the causal association between sarcopenia and the risk of AF, and the results of existing studies are inconsistent [3, 9, 10].

Low appendicular skeletal muscle mass (ASM) is an important clinical characteristic of sarcopenia, which has been recommended as a crucial diagnostic criterion for sarcopenia by the Asian Working Group for Sarcopenia (AWGS) and European Working Group on Sarcopenia in Older People (EWGSOP) [6, 7, 12]. Skeletal muscle is the largest organ in the human body and a primary site for glucose uptake and deposition [6, 10, 13]. The progressive loss of skeletal muscle mass not only leads to frailty, which are well defined as being associated with AF [2], but also increases the level of insulin resistance (IR) reaction and the risk of metabolic cardiac diseases [6, 13]. Skeletal muscle loss in older individuals has been demonstrated to be mainly due to loss of Type II muscle fibres, which are less sensitive to insulin action than Type I fibres [10, 11]. In addition, skeletal muscle loss cause a paucity of myokines secretion [11] and mitochondrial dysfunction [3, 10]. Myokines secreted by skeletal muscle prevent IR [11], while impaired mitochondrial function in skeletal muscle triggers systemic IR [3].

IR is a pathological condition in which tissues fail to respond normally to insulin [14, 15], and it has been suggested as an important risk factor for incident cardiovascular diseases including AF [14, 15]. However, it is unclear that the role of IR in the association between muscle mass loss and AF risk.

The measure of estimated glucose disposal rate (eGDR) has emerged as a novel and simple surrogate marker of IR, based on glycated haemoglobin (HbA1c), hypertension and waist circumference (WC) [16, 17, 18]. Its accuracy, reliability and clinical utility have been validated by the hyperinsulinaemic–euglycaemic clamp method, the gold standard for identifying IR in individuals with diabetes [16, 17, 19, 20]. Compared with hyperinsulinaemic–euglycaemic clamp and homeostasis model assessment for IR, eGDR is more appropriate for large‐scale and population‐based epidemiological investigations [16, 17]. Evidence has shown that low eGDR is associated with an increased risk of cardiovascular diseases, stroke, chronic kidney disease and mortality [16, 17, 19, 21].

The major objectives of this study were to investigate the associations between the collective effects of ASMI and eGDR, as assessed using baseline measurements and dual‐trajectory model, and AF risk in non‐diabetic older individuals based on a prospective cohort study.

## 2 Materials and Methods

### 2.1 Study Design and Participants

**FIGURE 1:**
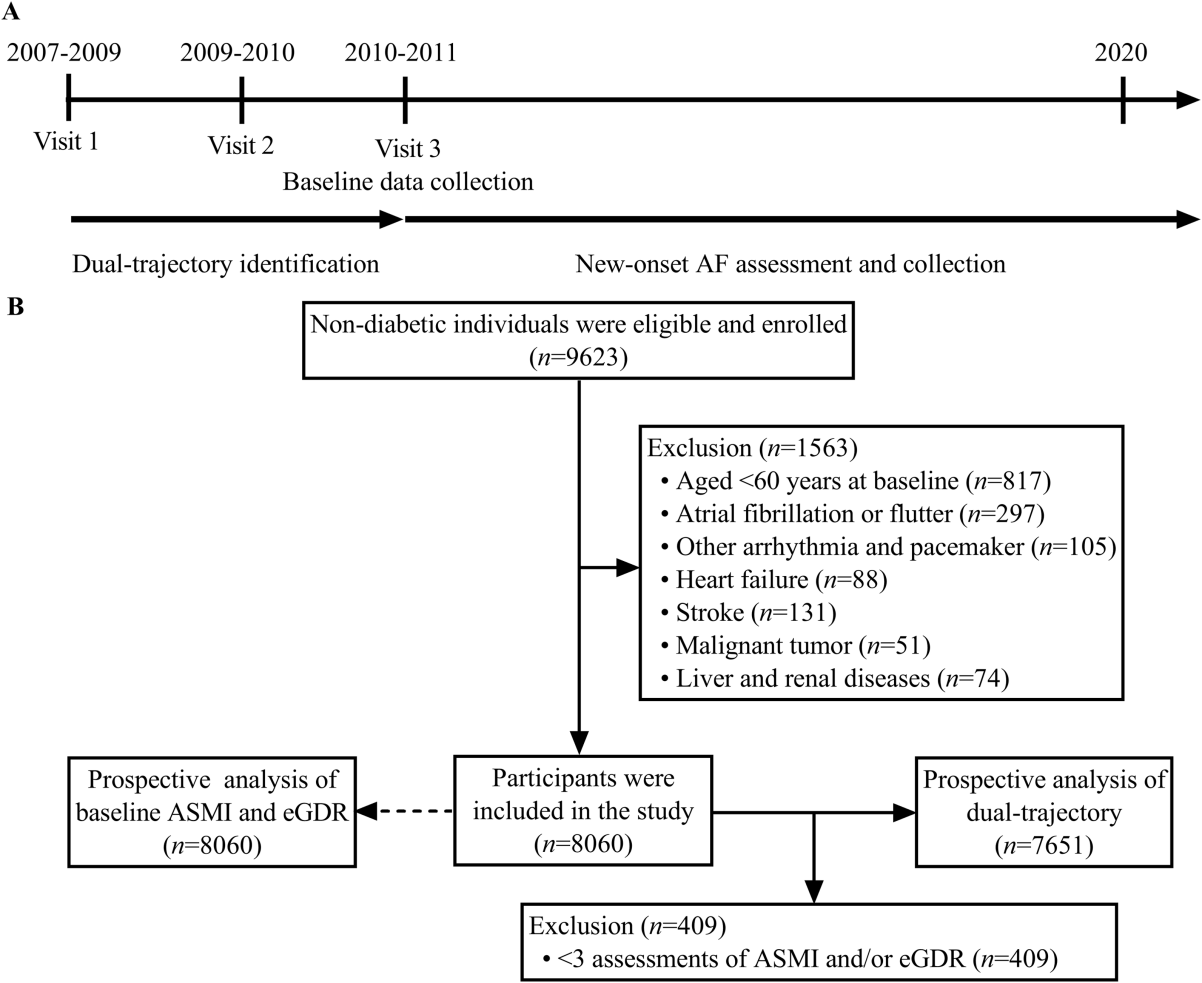
Flowchart of the study protocol. (A) Study design for examining the association of baseline and dual trajectory of ASMI and eGDR with AF risk. (B) Flowchart for the selection of study participants.

This longitudinal study used data from a community‐based prospective cohort study (registration number: ChiCTR‐EOC‐17013598) that aimed to elucidate the multiple risk factors associated with chronic diseases, as elsewhere [4, 22, 23]. A total of 8060 individuals aged 60 years and older were eligible and enrolled in this study. The initial survey (Visit 1) was from 2007 to 2009 and aided by the help of family physicians. Exclusions were made for participants who were diabetic (fasting plasma glucose [FPG] ≥ 7 mmol/L, HbA1c ≥ 6.5% or with antidiabetic agents); had AF or atrial flutter, heart failure, myocardial infarction, stroke, obstructive sleep apnoea syndrome, liver diseases and renal dysfunction (estimated glomerular filtration rate [eGFR] < 60 mL/min/1.73 m^2^) [24]; or those who lacked complete data for ASMI, WC, HbA1c and hypertension (yes or no) for the trajectory identification. After the initial survey, two waves of follow‐up were conducted from 2009 to 2010 (Visit 2) and 2010 to 2011 (Visit 3, the baseline for this study). Dual trajectory for ASMI and eGDR was identified using the data from Visits 1–3. The outcomes of interest were collected after Visit 3 and ended in 2020. The flowchart for this protocol is detailed in Figure 1.

This study was conducted in compliance with the Declaration of Helsinki and adhered to the Strengthening the Reporting of Observational Studies in Epidemiology (STROBE) reporting guidelines. The protocols were approved by the Research Ethics Committee of the Institute of Basic Medicine, Shandong Academy of Medical Sciences, and written informed consent was obtained from all participants.

### 2.2 Anthropometric and ASM Assessment

The assessments were conducted in a quiet and temperature‐controlled room (22°C–24°C) by trained research assistants who were blinded to the participants' clinical data. Height was measured using a wall‐mounted stadiometer and were to an accuracy of 0.1 cm. WC was measured in the late exhalation phase at the midpoint between the lower rib margin and the iliac crest using an inelastic tape to an accuracy of 0.1 cm.

Because of its accuracy and reliability, a multifrequency bioelectrical impedance analysis (BIA) device was used for evaluating loss of ASM [6, 7, 25]. In this study, all assessments of ASM were conducted by trained research assistants using a segmental multifrequency BIA device (InBody720, Biospace, Seoul, Korea) following the manufacturer's instructions. After fasting for at least 2 h and emptying the bladder, participants were asked to wear only underwear without shoes and were instructed to hold the electrode while standing barefoot on the other electrode of the device platform for 3–5 min to measure the ASM (kg) and body weight. The ASM index (ASMI) was calculated using the following formula: ASM (kg) divided by the square of the height (m). Participants with ASMI less than 7.0 kg/m^2^ for men and 5.7 kg/m^2^ for women were identified as the low ASMI according to AWGS criterion for sarcopenia [7, 12].

### 2.3 eGDR

The formula for eGDR has been described elsewhere [17, 18]: eGDR (mg/kg/min) = 21.158 − (0.09 × WC) − (3.407 × hypertension) − (0.551 × HbA1c) (WC [cm], hypertension [yes = 1/no = 0] and HbA1c [%]). Hypertension was defined as a systolic blood pressure (SBP) ≥ 140 mm Hg and/or a diastolic blood pressure (DBP) ≥ 90 mm Hg or treatment with antihypertensive medication. HbA1c was measured by ion‐exchange high‐performance liquid chromatography.

### 2.4 Covariates

Covariates included age, smoking status, alcohol consumption, physical activity, history of hypertension and dyslipidaemia, medication use for antihypertension and antidyslipidaemia, chronic obstructive pulmonary disease (COPD), blood pressure, FPG, plasma lipids, eGFR, common carotid artery intima–media thickness (CCA‐IMT) and plaque and anaemia (detailed in the Supporting Information).

### 2.5 Outcomes

The primary outcome for this study was the first occurrence of AF during the follow‐up period. AF was diagnosed according to the ICD‐10 code I48 (I48.0–I48.9) at hospitalization, specialized outpatient visits or through standard 12‐lead electrocardiograms. Among the new‐onset AF, persistent and paroxysmal AF were classified according to the episodes of AF sustain. The episodes of AF that sustain at least 7 days or require cardioversion to restore sinus rhythm were diagnosed as persistent AF, and the episodes of AF that sustain at least 1 day but spontaneously terminate within 7 days were diagnosed as paroxysmal AF [26]. Diagnoses of AF were adjudicated by a committee based on the review of medical records including electrocardiograms and medications.

### 2.6 Statistical Analyses

Baseline characteristics were presented as mean ± standard deviation (SD) or median with interquartile range (IQR; 25th–75th percentile) for continuous variables, according to the normality of the distribution determined by the Kolmogorov–Smirnov test and as numbers with percentage frequency for categorical variables. Differences in continuous variables were analysed using an independent sample *t* test or Mann–Whitney *U* test between two groups and a one‐way analysis of variance or Kruskal–Wallis test with post hoc Bonferroni tests among more than two groups, according to the normality of the distribution. Differences in categorical variables were analysed using the chi‐square test.

First, we assessed the association between the collective effects of baseline ASMI and eGDR and incident AF. Restricted cubic splines analyses were conducted to explore the dose–response association of baseline ASMI and eGDR with incident AF. Fine–Gray competing risk models were carried out to assess the interaction between continuous and categorical measurements of ASMI and eGDR on AF risk. Models were adjusted for the covariates including age, sex, smoking status, alcohol consumption, exercise, SBP, DBP, heart rate, plasma lipids, FPG, history of dyslipidaemia and medication for antihypertension and antidyslipidaemia, COPD, eGFR, haemoglobin and CCA‐IMT and plaque.

Second, we explored the association between the dual trajectory of ASMI and eGDR with the incidence of AF. A group‐based dual‐trajectory analysis model based on a semiparametric approach was used to identify the temporal progression of ASMI and eGDR in this study [27, 28, 29, 30]. This method allows insight into the association between the dynamics of ASMI and eGDR that evolve contemporaneously. Specifically, it evaluates the likelihood of eGDR trajectories following a specific ASMI trajectory, without presupposing a premise. In this model, first, the number of optimal trajectory groups was determined for inclusion into the model, ranging from two to six clusters. Then, the order of trajectory polynomials was adjusted, and the shapes of the trajectories were specified (linear, quadratic or cubic). The optimal dual‐trajectory model for ASMI and eGDR levels was determined according to the following criteria: the smallest Bayesian information criterion (BIC) value, the smallest Akaike's information criterion (AIC) value, higher average posterior probability (APP, more than 0.7), the smallest log‐likelihood value, maximum Entropy and no less than 5% of group membership. Fine–Gray competing risk models were conducted to assess the adjusted differences in incident AF among groups classified by optimal group‐based dual trajectories of ASMI and eGDR, using Group 4 (characterized by moderate‐stable ASMI and moderate‐stable eGDR) as the reference group. Fine–Gray competing risk models were adjusted for confounders including in the prospective analysis of baseline ASMI and eGDR and baseline ASMI and eGDR. Death from unknown cause was collected after Visit 3, which was used for identifying the dual trajectories of ASMI and eGDR (Figure 1).

To study potential heterogeneity, stratified analyses were conducted by age (< 70 vs. ≥ 70 years), sex (male vs. female), physical activity (no vs. yes), BMI (< 28.0 vs. ≥ 28.0 kg/m^2^) [31], eGFR (< 90 vs. ≥ 90 mL/min/1.73 m^2^) and anaemia (no vs. yes, cut‐off of haemoglobin was 130 g/L for men and 120 g/L for women [32]). The potential interactions between stratified variables and ASMI and eGDR baseline measurements, and dual trajectory was explored.

Sensitivity analyses were performed to test the robustness of the findings in this study. First, all analyses were performed separately for total, male and female participants. Second, the analysis models were adjusted for baseline conditions. Third, a Fine–Gray competing risk model was performed treating death from unknown causes as competing events. Fourth, a mediation analysis using SPSS Hayes process (Version 4.0) was conducted to assess the role of IR on the association between the loss of muscle mass and AF risk. Stratified analyses were also conducted to evaluate the effects of age, physical activity, BMI, renal dysfunction and anaemia.

The statistical analyses were conducted using SPSS (Version 26.0; IBM Corp., Armonk, NY, USA) and R software for analysis (Version 4.4.1; R Foundation for Statistical Computing, http://www.R‐project.org) and two‐sided *p* values of < 0.05 were set as statistically significant.

## 3 Results

### 3.1 Association Between Baseline ASMI, eGDR and Their Interaction and AF Incidence

A total of 8060 non‐diabetic participants were included in this prospective analysis of the associations between the collective effects of baseline ASMI and eGDR and incident AF (Figure 1). Among them, 4046 (50.20%) were female, 4014 (49.80%) were male and the mean age was 68.04 (SD: 6.12) years. Table 1 details the demographic and baseline characteristics of participants grouped by baseline ASMI and eGDR. Alcohol consumption, anaemia, WC, BMI and the levels of SBP, TCHO and FPG were higher, but the level of eGFR was lower in the low ASMI and low eGDR groups when compared to those in the normal ASMI and higher eGDR groups, respectively (all *p* < 0.05).

**TABLE 1:** Demographic and baseline characteristics of participants grouped by baseline ASMI and eGDR (*n* = 8060).

	Overall (*n* = 8060)	Grouped by baseline ASMI			Grouped by the tertile of baseline eGDR			
		Normal ASMI (*n* = 6321)	Low ASMI (*n* = 1739)	*p*	Low eGDR (*n* = 2592)	Moderate eGDR (*n* = 2682)	High eGDR (*n* = 2786)	*p*
Age, years	68.04 ± 6.12	67.87 ± 6.18	68.08 ± 6.10	0.189	68.08 ± 6.16	68.08 ± 6.05	67.96 ± 6.14	0.678
Sex, *n* (%)				< 0.001				< 0.001
Female	4046 (50.2)	3464 (54.8)	582 (33.5)		1106 (42.7)	1472 (54.9)^*^	1468 (52.7)^*,^, ^†^	
Male	4014 (49.8)	2857 (45.2)	1157 (66.5)		1486 (57.3)	1210 (45.1)	1318 (47.3)	
Smoking, *n* (%)	2297 (28.5)	1674 (26.5)	623 (35.8)	< 0.001	757 (29.2)	734 (27.4)	806 (28.9)	0.276
Alcohol consumption, *n* (%)	2837 (35.2)	2111 (33.4)	726 (41.7)	< 0.001	973 (37.5)	937 (34.9)^*^	927 (33.3)^*^	0.004
Physical exercise, *n* (%)	4560 (56.6)	3572 (56.5)	988 (56.9)	0.821	1473 (56.8)	1548 (57.7)	1539 (55.2)	0.173
WC, cm	81.28 ± 9.44	80.95 ± 9.43	82.48 ± 9.38	< 0.001	89.93 ± 6.23	77.68 ± 5.30^*^	76.69 ± 9.57^*,^ ^†^	< 0.001
BMI, kg/m^2^	24.03 ± 3.48	24.00 ± 3.50	24.15 ± 3.42	0.096	24.78 ± 3.53	23.64 ± 3.44^*^	23.70 ± 3.37^*^	< 0.001
SBP, mm Hg	146.53 ± 18.61	145.08 ± 18.08	146.92 ± 18.73	< 0.001	151.67 ± 18.02	149.09 ± 17.82^*^	139.09 ± 17.53^*,^, ^†^	< 0.001
DBP, mm Hg	70.77 ± 9.00	70.76 ± 9.00	70.81 ± 8.98	0.866	72.54 ± 9.04	71.76 ± 8.70^*^	68.18 ± 8.64^*,^, ^†^	< 0.001
Heart rate, beats/min	73.60 ± 8.84	73.62 ± 8.84	73.53 ± 8.85	0.718	74.44 ± 8.91	73.60 ± 8.66^*^	72.81 ± 8.88^*,^, ^†^	< 0.001
TCHO, mmol/L	4.94 ± 0.82	4.90 ± 0.80	4.96 ± 0.82	0.015	4.99 ± 0.85	4.93 ± 0.82^*^	4.92 ± 0.79^*^	0.004
Triglycerides, mmol/L	1.56 ± 0.57	1.55 ± 0.57	1.57 ± 0.57	0.404	1.62 ± 0.61	1.56 ± 0.56^*^	1.52 ± 0.55^*,^, ^†^	< 0.001
HDL‐c, mmol/L	1.27 ± 0.41	1.28 ± 0.42	1.26 ± 0.40	0.073	1.28 ± 0.42	1.28 ± 0.41	1.26 ± 0.41^*^	0.033
LDL‐c, mmol/L	2.96 ± 0.73	2.94 ± 0.70	2.96 ± 0.73	0.154	2.97 ± 0.76	2.94 ± 0.71	2.97 ± 0.72	0.323
FPG, mmol/L	5.61 ± 0.71	5.59 ± 0.71	5.71 ± 0.72	< 0.001	5.85 ± 0.66	5.49 ± 0.70^*^	5.51 ± 0.71^*^	< 0.001
HbA1c, %	5.45 ± 0.55	5.43 ± 0.56	5.53 ± 0.54	< 0.001	5.72 ± 0.44	5.35 ± 0.54^*^	5.30 ± 0.57^*,^, ^†^	< 0.001
Hypertension, *n* (%)	6066 (75.3)	4774 (75.5)	1292 (74.3)	0.292	2086 (80.5)	2018 (75.2)^*^	1962 (70.4)^*,^, ^†^	< 0.001
Dyslipidaemia, *n* (%)	2921 (36.2)	2261 (35.8)	660 (38.0)	0.094	979 (37.8)	983 (36.7)	959 (34.4)^*^	0.033
Antihypertensive medication, *n* (%)	4398 (54.6)	3464 (54.8)	934 (53.7)	0.418	1389 (53.6)	1455 (54.3)	1554 (55.8)	0.251
Antidyslipidaemia medication, *n* (%)	453 (5.6)	346 (5.5)	107 (6.2)	0.276	146 (5.6)	180 (6.7)	127 (4.6)^*^	0.003
**COPD, *n* (%)**	**733 (9.0)**	**550 (8.7)**	**183 (10.5)**	**0.019**	**265 (10.2)**	**236 (8.7)**	**232 (8.3)** ^*^	**0.044**
Haemoglobin, g/L	126.51 ± 8.55	126.56 ± 8.58	126.34 ± 8.41	0.338	126.22 ± 8.84	126.80 ± 8.52^*^	126.50 ± 8.29	0.050
Anaemia, *n* (%)	2140 (26.55)	1604 (25.38)	536 (30.82)	< 0.001	1009 (38.93)	594 (22.15)	537 (19.27)	< 0.001
**CCA‐IMT, mm**	**1.28 ± 0.56**	**1.27 ± 0.55**	**1.29 ± 0.56**	**0.186**	**1.33 ± 0.59**	**1.28 ± 0.54** ^*^	**1.25 ± 0.55** ^*^	**< 0.001**
**CCA‐plaque, *n* (%)**	**2088 (25.91)**	**1637 (25.90)**	**451 (25.93)**	**0.975**	**783 (30.21)**	**677 (25.24)** ^*^	**628 (22.54)** ^*^, ^†^	**< 0.001**
eGFR, mL/min/1.73 m^2^	92.08 ± 6.39	92.35 ± 6.43	91.12 ± 6.16	< 0.001	87.31 ± 4.50	93.51 ± 5.03*	95.15 ± 6.52^*,^, ^†^	< 0.001
ASMI, kg/m^2^	7.16 ± 0.91	7.46 ± 0.70	6.05 ± 0.70	< 0.001	6.95 ± 0.90	7.16 ± 0.92*	7.36 ± 0.91^*,^, ^†^	< 0.001
eGDR, mg/kg/min	8.29 ± 1.80	8.32 ± 1.80	8.16 ± 1.81	0.001	6.50 ± 0.58	7.84 ± 0.36*	10.38 ± 1.20^*,^, ^†^	< 0.001

*Note:* Results are mean ± SD or frequencies with percentages.

Abbreviations: ASMI, appendicular skeletal muscle mass index; BMI, body mass index; CCA‐IMT, common carotid artery intima–media thickness; COPD, chronic obstructive pulmonary disease; DBP, diastolic blood pressure; eGDR, estimated glucose disposal rate; eGFR, estimated glomerular filtration rate; FPG, fasting plasma glucose; HDL‐c, high‐density lipoprotein cholesterol; LDL‐c, low‐density lipoprotein cholesterol; SBP, systolic blood pressure; TCHO, total cholesterol; WC, waist circumference.

^*^ *p* < 0.05, compared with low eGDR group.

^†^ *p* < 0.05, compared with moderate eGDR group.

**FIGURE 2:**
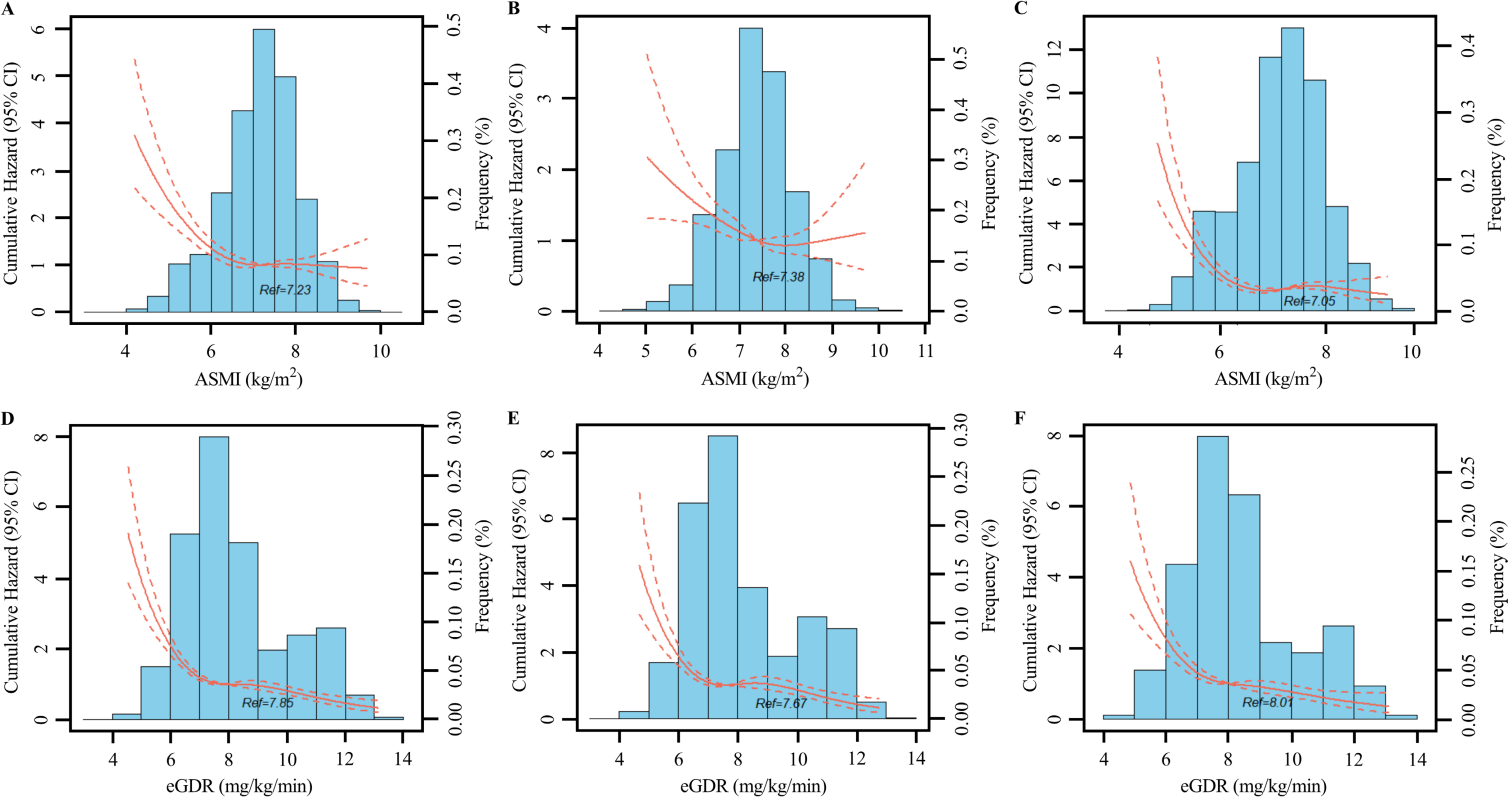
Dose–response association of baseline ASMI and eGDR with AF risk analysed using restricted cubic splines model. The association between continuous measurement ASMI and AF risk in total participants (A), male (B) and female (C). The association between continuous measurement eGDR and AF risk in total participants (D), male (E) and female (F). ASMI indicates appendicular skeletal muscle mass index; eGDR, estimated glucose disposal rate.

Over an average of 9.70 (IQR: 9.50–10.10) years of follow‐up, 888 (11.66 per 1000 person‐years) participants developed new‐onset AF. Restricted cubic splines analysis showed L‐shaped associations between AF risk and ASMI and eGDR in total participants, male and female. ASMI inflection points were 7.23, 7.38 and 7.05 kg/m^2^, and eGDR inflection points were 7.85, 7.67 and 8.01 mg/kg/min in total participants, male and female, respectively (Figure 2). When ASMI lower than inflection points, AF risk decreased by 18.0% (hazard ratio [HR]: 0.820, 95% confidence interval [CI]: 0.769–0.874), 14.7% (HR: 0.853, 95% CI: 0.769–0.945) and 24.7% (HR: 0.753, 95% CI: 0.676–0.839) with one‐SD AMSI increment in total participants, male and female, respectively, after adjustment for confounders (*p*_adj._ < 0.001; Table S1). When eGDR lower than inflection points, AF risk decreased by 36.3% (HR: 0.637, 95% CI: 0.586–0.692), 24.9% (HR: 0.751, 95% CI: 0.687–0.822) and 36.5% (HR: 0.635, 95% CI: 0.556–0.724) with one‐SD eGDR increment in total participants, male and female, respectively (*p*_adj._ < 0.001; Table S1).

After participants were classified into normal and low ASMI groups, AF risk in the low ASMI group increased by 0.76‐, 0.42‐ and 1.56‐fold in total participants, male and female when compared to the normal ASMI group, respectively (*p*_adj._ < 0.001; Figure S1 and Table S2).

Interaction analysis showed that there was a synergistic effect of ASMI and eGDR on AF risk in total participants, male and female (Table S3). The results were similar after AF was classified as paroxysmal and persistent AF (Tables S1–S3 and Figures S1 and S2).

### 3.2 Association Between the Dual Trajectory of ASMI and eGDR and AF Incidence

**FIGURE 3:**
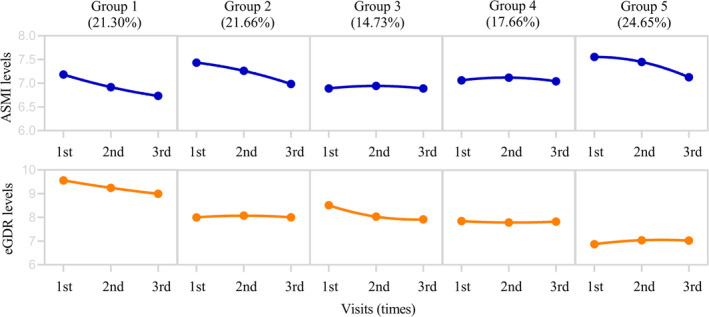
Dual trajectories of ASMI and eGDR identified using group‐based dual‐trajectory modelling. Dots show group‐specific mean observed levels while solid lines represent the best fitted trajectories. ASMI and eGDR were modelled as a function of follow‐up time. Group 1 was high‐decrease ASMI and high‐decrease eGDR; Group 2, high‐decrease ASMI and moderate‐stable eGDR; Group 3, moderate‐stable ASMI and moderate‐decrease eGDR; Group 4, moderate‐stable ASMI and moderate‐stable eGDR; Group 5, high‐decrease ASMI and low‐stable eGDR. ASMI indicates appendicular skeletal muscle mass index; eGDR, estimated glucose disposal rate.

Among the 8060 participants, 7651 were eligible and were included in the dual‐trajectory analysis of ASMI and eGDR (Figure 1). There were 3835 (50.12%) women and 3816 (49.88) men, and the mean age was 68.09 (SD: 6.18) years. Five distinct dual trajectories of ASMI and eGDR were identified, denoted as Group 1 (high‐decrease ASMI and high‐decrease eGDR, *n* = 1630, 21.30%), Group 2 (high‐decrease ASMI and moderate‐stable eGDR, *n* = 1657, 21.66%), Group 3 (moderate‐stable ASMI and moderate‐decrease eGDR, *n* = 1127, 14.73%), Group 4 (moderate‐stable ASMI and moderate‐stable eGDR, *n* = 1351, 17.66%) and Group 5 (high‐decrease ASMI and low‐stable eGDR, *n* = 1886, 24.65%), as detailed in Figure 3. The BIC, AIC and the average of APP for these five types were 78 868.31, 78 817.53 and 0.80 (Table S4). Table 2 shows the demographic and baseline characteristics of the non‐diabetic participants grouped by the dual trajectory of ASMI and eGDR. Participants in Group 5 were more likely to have hypertension, dyslipidaemia, using antihypertensive medications and anaemia and had higher levels of WC, BMI, SBP, DBP, FPG and HbA1c but lower level of eGFR.

**TABLE 2:** Demographic and baseline characteristics of participants grouped by dual trajectory of ASMI and eGDR (*n* = 7651).

	Overall (*n* = 7651)	Group 1 (*n* = 1630)	Group 2 (*n* = 1657)	Group 3 (*n* = 1127)	Group 4 (*n* = 1351)	Group 5 (*n* = 1886)	*p*
Age, years	68.09 ± 6.18	68.09 ± 6.26	68.43 ± 6.35	67.67 ± 5.95^*^	68.25 ± 6.26	67.95 ± 6.01	0.017
Sex, *n* (%)							< 0.001
Female	3835 (50.1)	791 (48.5)	865 (52.2)^*^	535 (47.5)^†^	796 (58.9)^*,^, ^†,^, ^‡^	848 (45.0)^*^, ^†^, ^⁋^	
Male	3816 (49.9)	839 (51.5)	792 (47.8)	592 (52.5)	555 (41.1)	1038 (55.0)	
Smoking, *n* (%)	2205 (28.8)	505 (31.0)	480 (29.0)	362 (32.1)	330 (24.4)^*,^, ^†^, ^‡^	528 (28.0)^‡^, ^⁋^	< 0.001
Alcohol consumption, *n* (%)	2715 (35.5)	578 (35.5)	595 (35.9)	408 (36.2)	443 (32.8)	691 (36.6)	0.217
Physical exercise, *n* (%)	4384 (57.3)	882 (54.1)	970 (58.5)^*^	639 (56.7)	773 (57.2)	1120 (59.4)^*^	0.023
WC, cm	81.46 ± 9.53	80.82 ± 8.85	78.10 ± 9.77*	81.94 ± 8.99^*^, ^†^	78.99 ± 9.93^*^, ^†^, ^‡^	86.45 ± 7.63^*^, ^†^, ^‡^, ^⁋^	< 0.001
BMI, kg/m^2^	24.00 ± 3.53	23.85 ± 3.47	23.85 ± 3.59	24.13 ± 3.53	23.73 ± 3.48^‡^	24.40 ± 3.52^*^, ^†^, ^‡^, ^⁋^	< 0.001
SBP, mm Hg	147.30 ± 18.68	141.29 ± 18.43	149.10 ± 18.24*	143.83 ± 19.48^*^, ^†^	149.82 ± 17.60^*^, ^‡^	151.18 ± 17.90^*^, ^†^, ^‡^, ^⁋^	< 0.001
DBP, mm Hg	70.94 ± 9.06	68.78 ± 8.84	71.79 ± 8.94*	69.56 ± 9.20^*^, ^†^	71.42 ± 8.80^*^, ^‡^	72.56 ± 8.99^*^, ^†^, ^‡^, ^⁋^	< 0.001
Heart rate, beats/min	73.83 ± 8.29	73.36 ± 8.68	73.89 ± 8.89	73.51 ± 8.84	73.86 ± 8.64	74.34 ± 9.01^*^	0.014
TCHO, mmol/L	4.95 ± 0.83	4.95 ± 0.81	4.95 ± 0.83	4.94 ± 0.81	4.97 ± 0.84	4.95 ± 0.94	0.931
Triglycerides, mmol/L	1.57 ± 0.58	1.55 ± 0.59	1.56 ± 0.57	1.54 ± 0.57	1.56 ± 0.56	1.59 ± 0.59	0.147
HDL‐c, mmol/L	1.28 ± 0.42	1.26 ± 0.41	1.29 ± 0.42	1.28 ± 0.43	1.29 ± 0.41	1.28 ± 0.42	0.534
LDL‐c, mmol/L	2.96 ± 0.74	2.98 ± 0.74	2.95 ± 0.72	2.96 ± 0.73	2.97 ± 0.73	2.94 ± 0.75	0.534
FPG, mmol/L	5.61 ± 0.71	5.63 ± 0.71	5.50 ± 0.71*	5.59 ± 0.70^*^, ^†^	5.56 ± 0.71^*^, ^†^, ^‡^	5.74 ± 0.67^*^, ^†^, ^‡^, ^⁋^	< 0.001
HbA1c, %	5.47 ± 0.56	5.47 ± 0.56	5.33 ± 0.58*	5.48 ± 0.55^*^, ^†^	5.40 ± 0.58^*^, ^†^, ^‡^	5.64 ± 0.48^*^, ^†^, ^‡^, ^⁋^	< 0.001
Hypertension, *n* (%)	5877 (76.8)	639 (39.2)	1558 (94.0)	527 (46.8)	1280 (94.7)	1873 (99.3)	< 0.001
Dyslipidaemia, *n* (%)	2738 (35.8)	561 (34.4)	580 (35.0)	380 (33.7)	503 (37.2)	714 (37.9)	0.071
Antihypertensive medication, *n* (%)	4217 (55.1)	467 (28.7)	1079 (65.1)*	378 (33.5)^*^, ^†^	917 (67.9)*, ^‡^	1376 (73.0)*, ^†^, ^‡^, ^⁋^	< 0.001
Antidyslipidaemia medication, *n* (%)	390 (5.1)	61 (3.7)	78 (4.7)	54 (4.8)	86 (6.4)^*^, ^†^	111 (5.9)^*^	0.008
**COPD, *n* (%)**	**679 (8.8)**	**135 (8.2)**	**140 (8.4)**	**107 (9.4)**	**121 (8.9)**	**176 (9.3)**	**0.715**
Haemoglobin, g/L	126.58 ± 8.58	127.12 ± 8.41	128.39 ± 8.29*	126.54 ± 8.54^†^	127.32 ± 8.29^†^	124.00 ± 8.63^*^, ^†^, ^‡^, ^⁋^	< 0.001
Anaemia, n (%)	2030 (26.53)	398 (24.42)	339 (20.46)*	324 (28.75)^*^, ^†^	290 (21.47)^*^, ^‡^	679 (36.00)^*^, ^†^, ^‡^, ^⁋^	< 0.001
**CCA‐IMT, mm**	**1.28 ± 0.56**	**1.28 ± 0.57**	**1.28 ± 0.56**	**1.26 ± 0.55**	**1.28 ± 0.53**	**1.31 ± 0.58**	**0.152**
**CCA‐plaque, *n* (%)**	**1978 (25.9)**	**397 (24.4)**	**420 (25.3)**	**298 (26.4)**	**349 (25.8)**	**514 (27.3)**	**0.372**
eGFR, mL/min/1.73 m^2^	91.80 (87.62–96.27)	92.64 (88.58–96.91)	93.79 (89.13–98.44)*	91.78 (87.77–96.24)^*^, ^†^	92.91 (88.82–97.71)^‡^	89.17 (85.52–92.47)^*^, ^†^, ^‡^, ^⁋^	< 0.001
ASMI, kg/m^2^							
Visit 1	7.17 ± 0.90	7.18 ± 0.45	7.44 ± 0.41*	6.89 ± 0.48^*^, ^†^	7.07 ± 0.46^*^, ^†^, ^‡^	7.56 ± 0.41^*^, ^†^, ^‡^, ^⁋^	< 0.001
Visit 2	7.06 ± 0.85	6.92 ± 0.39	7.27 ± 0.47*	6.95 ± 0.49^*^, ^†^	7.12 ± 0.41^*^, ^†^, ^‡^	7.45 ± 0.37^*^, ^†^, ^‡^, ^⁋^	< 0.001
Visit 3	7.04 ± 0.86	6.73 ± 0.51	6.99 ± 0.45*	6.89 ± 0.45^*^, ^†^	7.05 ± 0.40^*^, ^†^, ^‡^	7.13 ± 0.37^*^, ^†^, ^‡^, ^⁋^	< 0.001
eGDR, mg/kg/min							
Visit 1	8.21 ± 1.77	9.56 ± 0.55	8.01 ± 0.51*	8.52 ± 0.53^*^, ^†^	7.85 ± 0.48^*^, ^†^, ^‡^	6.88 ± 0.43^*^, ^†^, ^‡^, ^⁋^	< 0.001
Visit 2	8.02 ± 1.21	9.25 ± 0.46	8.08 ± 0.49*	8.04 ± 0.52^*^, ^†^	7.79 ± 0.52^*^, ^†^, ^‡^	7.04 ± 0.41^*^, ^†^, ^‡^, ^⁋^	< 0.001
Visit 3	8.01 ± 1.29	9.00 ± 0.48	8.01 ± 0.44*	7.92 ± 0.39^*^, ^†^	7.83 ± 0.41^*^, ^†^, ^‡^	7.03 ± 0.46^*^, ^†^, ^‡^, ^⁋^	< 0.001

*Note:* Results are mean ± SD, median with interquartile range or frequencies with percentages.

Abbreviations: ASMI, appendicular skeletal muscle mass index; BMI, body mass index; CCA‐IMT, common carotid artery intima–media thickness; COPD, chronic obstructive pulmonary disease; DBP, diastolic blood pressure; eGDR, estimated glucose disposal rate; eGFR, estimated glomerular filtration rate; FPG, fasting plasma glucose; HDL‐c, high‐density lipoprotein cholesterol; LDL‐c, low‐density lipoprotein cholesterol; SBP, systolic blood pressure; TCHO, total cholesterol; WC, waist circumference.

^*^ *p* < 0.05, compared with Group 1.

^†^ *p* < 0.05, compared with Group 2.

^‡^ *p* < 0.05, compared with Group 3.

^⁋^ *p* < 0.05, compared with Group 4.

**FIGURE 4:**
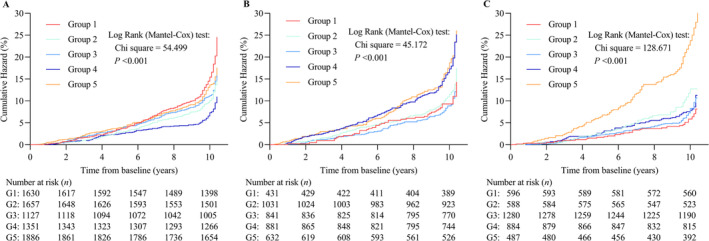
Cumulative hazard of AF in the groups classified by the dual trajectory of ASMI and eGDR. (A) Cumulative hazard of AF in total participants. Group 1 was high‐decrease ASMI and high‐decrease eGDR; Group 2, high‐decrease ASMI and moderate‐stable eGDR; Group 3, moderate‐stable ASMI and moderate‐decrease eGDR; Group 4, moderate‐stable ASMI and moderate‐stable eGDR; Group 5, high‐decrease ASMI and low‐stable eGDR. (B) Cumulative hazard of AF in male. Group 1 was high‐slight‐decrease ASMI and moderate‐stable eGDR; Group 2, moderate‐decrease ASMI and moderate‐stable eGDR; Group 3, moderate‐stable ASMI and moderate‐stable eGDR; Group 4, high‐significant‐decrease ASMI and low‐stable eGDR; Group 5, low‐decrease ASMI and low‐decrease eGDR. (B) Cumulative hazard of AF in female. Group 1 was high‐stable ASMI and high‐stable eGDR; Group 2, high‐decrease ASMI and low‐decrease eGDR; Group 3, moderate‐stable ASMI and high‐decrease eGDR; Group 4, moderate‐decrease ASMI and moderate‐stable eGDR; Group 5, low‐decrease ASMI and low‐decrease eGDR.

Over an average of 9.70 (IQR: 9.50–10.10) years follow‐up, 866 (11.97 per 1000 person‐years) participants developed new‐onset AF. Group 1 had the highest incidence of AF (16.05 per 1000 person‐years), while Group 4 exhibited the lowest incidence of AF (7.03 per 1000 person‐years, Table S5). Group 1 presented the highest risk of new‐onset AF (HR: 2.255, 95% CI: 1.769–2.876, *p*_adj._ < 0.001) when compared to Group 4 after adjustment for potential confounders including baseline ASMI and eGDR levels. This risk was followed by those in Group 5 (HR: 1.893, 95% CI: 1.491–2.403, *p*_adj._ < 0.001). Group 3 also had an elevated risk (HR: 1.630, 95% CI: 1.244–2.136, *p*_adj._ < 0.001), as did Group 2 (HR: 1.513, 95% CI: 1.171–1.955, *p*_adj._ = 0.001; Figure 4A and Table S5). When clinical subtypes were classified, similar association were found between the dual trajectory of ASMI and eGDR and persistent AF risk. Group 1 but not Groups 2, 3 and 5 associated with an increased risk of paroxysmal AF as detailed in Table S5 and Figure S3A,D.

We also separately analysed the effects of dual trajectories of ASMI and eGDR on the risk of overall, paroxysmal and persistent AF in male and female. Similar as the analysed results in total participants, five distinct dual trajectories of ASMI and eGDR were identified in male and female (Tables S4 and S5 and Figures S3 and S4).

### 3.3 Sensitivity and Stratification Analysis

**FIGURE 5:**
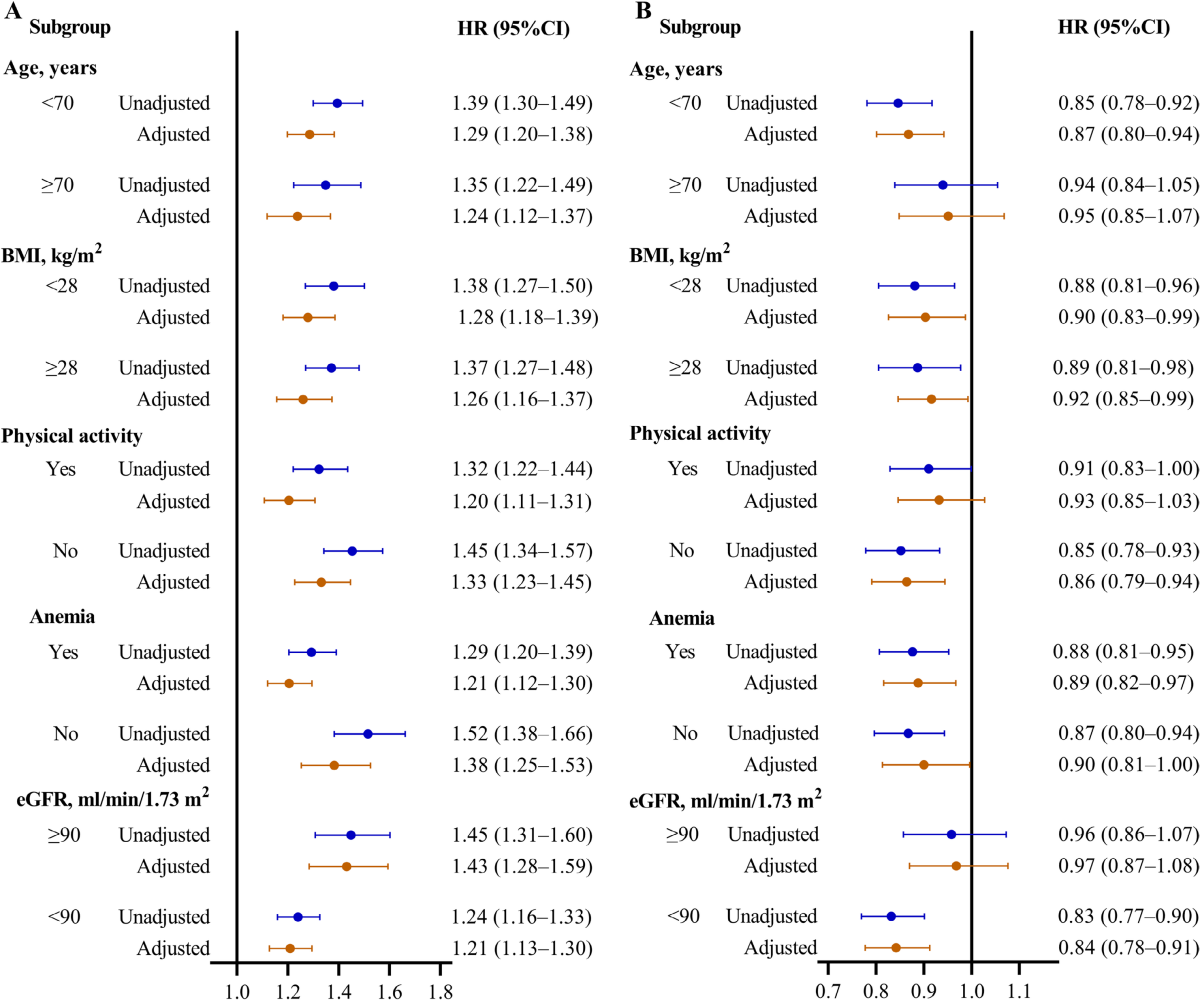
Stratified analysis of the association between the collective effect of baseline ASMI and eGDR and AF risk. (A) Association between collective effect of baseline ASMI and eGDR, assessed by categorical measurements, and AF risk. (B) Association between collective effect of baseline ASMI and eGDR, assessed by one‐SD increment, and AF risk. Adjusted models were adjusted for the covariates including age, sex, smoking status, alcohol consumption, exercise, SBP, DBP, heart rate, plasma lipids, FPG, history of dyslipidaemia and medications for antihypertension and antidyslipidaemia, COPD, eGFR, haemoglobin and CCA‐IMT and plaque. BMI indicates body mass index; eGFR, estimated glomerular filtration rate.

First, we performed subgroup analyses stratified by age, physical activity, BMI, eGFR and anaemia to explore the association between the collective effect of baseline ASMI and eGDR and AF risk. The associations of baseline ASMI and eGDR (Figure S5) and their interaction (Figure 5 and Table S6) with AF risk were consistent and stable across stratified variables.

Then, we assessed the direct effect of baseline ASMI and the indirect effect of baseline eGDR on the risk of AF using a mediation analysis. The results showed that baseline eGDR played a mediating role in the association between baseline ASMI and the risks of overall, paroxysmal and persistent AF in total participants, male and female (Figure S6).

**FIGURE 6:**
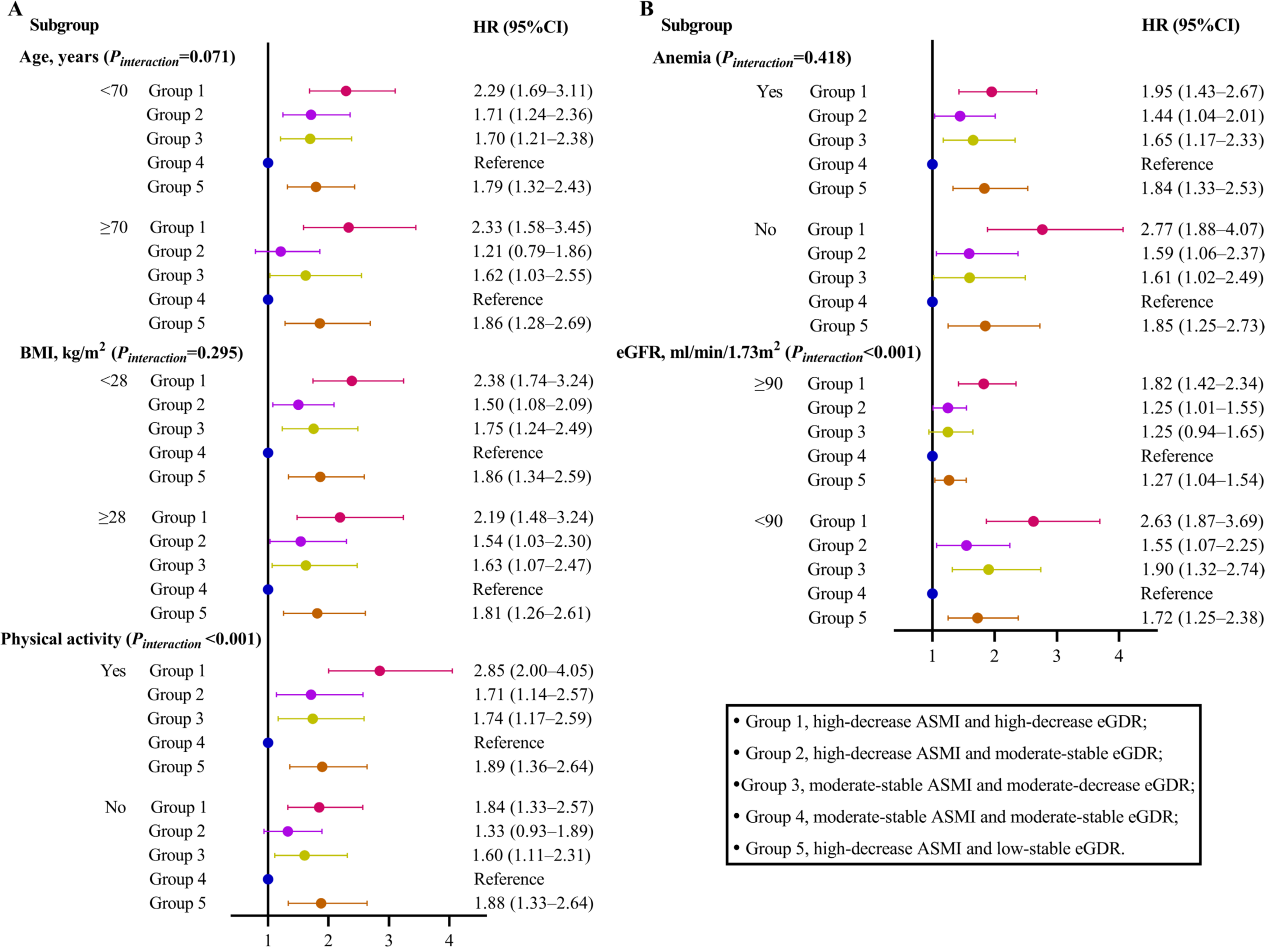
Stratified analysis of the association between dual trajectory of ASMI and eGDR and AF risk. Models were adjusted for the covariates including age, sex, smoking status, alcohol consumption, exercise, SBP, DBP, heart rate, plasma lipids, FPG, history of dyslipidaemia and medications for antihypertension and antidyslipidaemia, COPD, eGFR, haemoglobin and CCA‐IMT and plaque. Group 1 was high‐decrease ASMI and high‐decrease eGDR; Group 2, high‐decrease ASMI and moderate‐stable eGDR; Group 3, moderate‐stable ASMI and moderate‐decrease eGDR; Group 4, moderate‐stable ASMI and moderate‐stable eGDR; Group 5, high‐decrease ASMI and low‐stable eGDR. BMI indicates body mass index; eGFR, estimated glomerular filtration rate.

Next, we conducted subgroup analyses to assess the association between the dual trajectory of ASMI and eGDR and AF risk in detail. Across stratified variables, the association between dual‐trajectory types and AF risk was consistent and stable as demonstrated by the fact that Group 1, presented as the most prone to AF, followed by Group 5. The stratification analysis revealed that the dual trajectory of ASMI and eGDR was significantly associated with a higher risk of AF in the subgroups of participants younger than 70 years, BMI < 28 kg/m^2^, without anaemia (all *p*_interaction_ > 0.05). While the dual‐trajectory types exhibited a more pronounced predicting value among males, regular physical activity and eGFR < 90 mL/min/1.73 m^2^ (all *p*_interaction_ < 0.001, Figure 6).

The demographics and clinical characteristics of 409 participants, who were excluded from the dual‐trajectory analysis of ASMI and eGDR, are detailed in Table S7.

## 4 Discussion

The main findings of this study are that low ASMI and eGDR were independently associated with high risk of AF in non‐diabetic older individuals. Restricted cubic splines analysis demonstrated L‐shaped associations between AF risk and ASMI and eGDR. Baseline ASMI and eGDR exhibited a synergistic interplay for increased AF risk. Five distinct coevolutionary patterns of ASMI and eGDR over time were identified using dual‐trajectory analysis. Those non‐diabetic older individuals with high‐decrease ASMI and high‐decrease eGDR had the highest incidence of AF and the greatest increased risk of new‐onset AF when compared to those with moderate‐stable ASMI and moderate‐stable eGDR, who had the lowest incidence of AF. These findings remained robust after sensitivity and stratification analyses, particularly stratified by sex.

Although cross‐sectional studies demonstrated that sarcopenia was associated with an increased AF risk [9, 10], it is unclear as to the causal association between skeletal muscle mass loss and AF. Shim et al. [9] did not establish a causal association between skeletal muscle mass loss and AF risk in a cohort study, while Tang et al. [3] found that sarcopenia increased AF risk by 61% in a cohort study. Our data demonstrated that low ASMI group was associated with an 0.8‐fold increased risk of AF when compared to normal ASMI group. One‐SD ASMI increment was associated with an 18.0% decreased risk of AF. This association was still robust after subgroup analyses and adjustment for confounders. Despite the differences in age, race, region and sample size, the differences in the follow‐up duration may be an important cause for disagreement between these studies.

Low eGDR, a widely accepted surrogate marker of IR, is closely associated with an increased risk of cardiovascular diseases in diabetes and non‐diabetes [16, 17, 33]. In patients after radiofrequency catheter ablation, a decrease in eGDR leads to an increased risk of AF recurrence [34]. In this study, we found that AF risk decreased by 36.3% with one‐SD increment of eGDR.

We also found that the associations between baseline ASMI, eGDR and AF risk were nonlinear (L‐shaped). AF risk was steeply decreased with an ASMI and eGDR increment in participants with ASMI less than 7.23 kg/m^2^ and eGDR less than 7.85 mg/kg/min. However, AF risk was significantly reduced with ASMI and eGDR increments in those with ASMI and eGDR higher than these inflection points. The main reason for these results may be that there is a plateau in insulin sensitivity caused by high levels of ASM.

To investigate the collective effect of baseline ASMI and eGDR on AF risk, we performed an interaction analysis. The results showed that AF risk was increased by 14.5% in participants with low ASMI and moderate eGDR and 123.9% in those with low ASMI and low eGDR when compared to those with normal ASMI and high eGDR. With one‐SD increment in ASMI and eGDR, AF risk was decreased by 36.3%, even in stratification analysis and after adjustment for confounders. This indicated that there was a synergistic interaction between loss of skeletal muscle mass and IR on increased AF risk.

The progressive loss of skeletal muscle mass was accompanied by a chronic progression of IR [6, 13, 35]. To understand the association between AF risk and loss of skeletal muscle mass and IR in non‐diabetic older adults, we further determined the dynamic types of ASMI and eGDR that evolve contemporaneously using dual‐trajectory analysis and identified five dual trajectories of ASMI and eGDR types in this study. The risks of AF showed significant variability across different combinations of ASMI and eGDR trajectories. The participants in Group 1, with high‐decrease ASMI and high‐decrease eGDR, were at the highest risk of AF followed by Group 5 (with high‐decrease ASMI and low‐stable eGDR), However, Group 4, characterized by moderate‐stable ASMI and moderate‐stable eGDR, exhibited the lowest risk. This association remained robust with multiple sensitivity analyses including subgroup analyses and adjustment for potential confounders including baseline ASMI and eGDR. This indicated that a progressive loss of ASM and increase of IR can significantly and synergistically elevate AF risk in non‐diabetic older individuals, such as those in Group 1. Therefore, careful monitoring and strategic management of ASMI and eGDR trajectories are necessary to mitigate AF risk.

The dynamic assessment of ASMI and eGDR through three repeated measurements provides a comprehensive perspective that cannot be seen by a single baseline measurement. Evidence revealed the distinct advantage of the dual‐trajectory model, allowing for a more integrated examination of the connection between the trajectories of two related and contemporaneously evolving outcomes [27, 28, 30]. It summarizes multiple and dynamic associations between ASMI and eGDR across various trajectory patterns. By identifying distinct trajectory patterns, we conducted a deeper exploration of the collective effect between ASMI and eGDR. This offers valuable insight into the association between the evolution in ASMI and eGDR over time and AF risk within specific subgroups.

Skeletal muscle loss has been demonstrated to be associated with impaired left ventricular diastolic function, resulting in profibrillatory remodelling of the left atrium, and ultimately cause AF [3, 10, 11]. Although the underlying mechanism of skeletal muscle loss affecting AF is complicated [3], IR may be a crucial mediator or collaborator in the association between skeletal muscle loss and the risk of AF. As the main organ responsible for insulin‐induced glucose metabolism, progressive skeletal muscle loss was always accompanied with progressive paucity of myokines secretion [11], mitochondrial dysfunction [3, 10], adipose accumulation and ultimately causes IR. Given both skeletal muscle loss and IR are important risk factors for AF, this may be the reason why the participants who with progressive skeletal muscle mass loss and IR increasing, presented as high‐decrease ASMI and high‐decrease eGDR, exhibited a collective effect on the high risk of AF. It was verified by the results of mediation analyses and stratified analyses.

There are several factors influence the collective effect of skeletal muscle loss and IR on AF risk. In the stratified analysis, besides sex and age, we found that obesity, physical activity, anaemia and renal function modified the association between the collective effect of ASMI and eGDR and AF risk. These factors are all associated with skeletal muscle loss and IR. It indicates that proper managements of these factors are crucial for reducing AF risk in subjects with skeletal muscle loss and increased IR.

The associations between ASMI, eGDR and AF risk have been independently investigated in previous studies [3, 9, 10, 34]. Our study extended these findings by elucidating the compounded effect of ASMI and eGDR on AF risk. In this study, the results of dual‐trajectory analysis not only aligned with but also extended the findings from the prospective analysis of the association between baseline ASMI and eGDR and AF risk. Recognizing these patterns of dual trajectory in ASMI and eGDR can satisfactorily address the complex effect between ASMI and eGDR in a more targeted manner, which helps tailor intervention measures and support strategies to meet the unique needs of each group.

## 5 Strengths and Limitations

The major strengths of this study were that the association between ASMI and eGDR and incident AF was comprehensively assessed using baseline and dual trajectory of ASMI and eGDR. Our study contributed to a piece of important evidence on the dynamic interplay between ASMI and eGDR in AF risk, particularly in non‐diabetic older populations. Second, a Fine–Gray competing risk model was performed to limit the bias induced by death from unknown causes. In addition, the considerable sample size and prospective cohort design may also be a strength, as this enables robust longitudinal analysis.

However, several limitations should also be considered. First, the generalization of findings in this study may be limited because the participants were primarily recruited from the Shandong area of China, with the majority being of Han nationality. Second, we did not consider the participants' dietary balance, genetic risk or whether they suffer from sleep apnoea; these omissions may introduce bias in the study results. Lifestyle and genetic risk are associated with AF [36, 37] and skeletal muscle mass loss [7, 38]. Sleep apnoea is a common comorbidity in older individuals, contributing to AF by modulating the activity of the autonomic nervous system and remodelling atrial structure [39]. Third, this study did not include the potential impact of detailed medication use on fat loss and other comorbidities, which may lead to a gap in fully understanding these interrelationships. Finally, residual confounders may still affect the results due to the nature of observational design per se.

## 6 Conclusions

This longitudinal cohort study revealed a significant association between the collective effect of ASMI and eGDR and AF risk in non‐diabetic older individuals. The interplay of low ASMI and eGDR, as well as the temporal evolution of ASMI and eGDR decreasing over time, exhibits great effects on increased risk of new‐onset AF. Our study reinforces the roles of ASMI and eGDR as potential biomarkers for stratified risk management of AF. Collective management of skeletal muscle mass and IR might be a feasible and effective management strategy for preventing and controlling AF in non‐diabetic older adults. However, further studies are needed, including the study of other ethnicities, those with diabetes and in ages of less than 60 years, especially the clinical trials of combined intervention of muscle‐strengthening exercises, such as aerobic and/or resistance exercise, and pharmacologic agents to reduce IR.

## Conflicts of Interest

The authors declare no conflicts of interest.

## Supporting information


**Table S1.** Association between baseline ASMI and the incidence of AF.
**Table S2.** Different cumulative hazard of AF between the low and normal ASMI groups.
**Table S3.** Interaction between baseline ASMI and eGDR on the incidence of AF.
**Table S4.** Model fitting parameters for dual trajectories of ASMI and eGDR.
**Table S5.** Association between dual trajectory of ASMI and eGDR and the incidence of AF.
**Table S6.** Stratified analysis of the association between the collective effect of baseline ASMI and eGDR and AF risk.
**Table S7.** Demographic and baseline characteristics of participants excluded from analysis of dual trajectory of ASMI and eGDR (*n* = 409).
**Figure S1.** Cumulative hazard of AF between the low and normal ASMI groups. The cumulative hazard of overall AF in total participants (A), male (B) and female (C); the cumulative hazard of paroxysmal AF in total participants (D), male (E) and female (F); and the cumulative hazard of persistent AF in total participants (G), male (H) and female (I). ASMI indicates appendicular skeletal muscle mass index.
**Figure S2.** Dose–response association of baseline ASMI and eGDR with paroxysmal and persistent AF risk analysed using restricted cubic splines model. The association between continuous measurement ASMI and paroxysmal AF risk in total participants (A), male (B) and female (C). The association between continuous measurement ASMI and persistent AF risk in total participants (D), male (E) and female (F). The association between continuous measurement eGDR and paroxysmal AF risk in total participants (G), male (H) and female (I). The association between continuous measurement eGDR and persistent AF risk in total participants (J), male (K) and female (L). ASMI indicates appendicular skeletal muscle mass index; eGDR, estimated glucose disposal rate.
**Figure S3.** Cumulative hazard of paroxysmal and persistent AF in the groups classified by the dual trajectory of ASMI and eGDR. Cumulative hazard of paroxysmal AF in total participants (A), male (B) and female (C); cumulative hazard of persistent AF in total participants (D), male (E) and female (F). For total participants, Group 1 was high‐decrease ASMI and high‐decrease eGDR; Group 2, high‐decrease ASMI and moderate‐stable eGDR; Group 3, moderate‐stable ASMI and moderate‐decrease eGDR; Group 4, moderate‐stable ASMI and moderate‐stable eGDR; Group 5, high‐decrease ASMI and low‐stable eGDR. For male, Group 1 was high‐slight‐decrease ASMI and moderate‐stable eGDR; Group 2, moderate‐decrease ASMI and moderate‐stable eGDR; Group 3, moderate‐stable ASMI and moderate‐stable eGDR; Group 4, high‐significant‐decrease ASMI and low‐stable eGDR; Group 5, low‐decrease ASMI and low‐decrease eGDR. For female, Group 1 was high‐stable ASMI and high‐stable eGDR; Group 2, high‐decrease ASMI and low‐decrease eGDR; Group 3, moderate‐stable ASMI and high‐decrease eGDR; Group 4, moderate‐decrease ASMI and moderate‐stable eGDR; Group 5, low‐decrease ASMI and low‐decrease eGDR.
**Figure S4.** Dual trajectories of ASMI and eGDR identified in male and female using group‐based dual‐trajectory modelling. (A) Dual trajectories of ASMI and eGDR in male. Group 1 was high‐slight‐decrease ASMI and moderate‐stable eGDR; Group 2, moderate‐decrease ASMI and moderate‐stable eGDR; Group 3, moderate‐stable ASMI and moderate‐stable eGDR; Group 4, high‐significant‐decrease ASMI and low‐stable eGDR; Group 5, low‐decrease ASMI and low‐decrease eGDR. (B) Dual trajectories of ASMI and eGDR in female. Group 1 was high‐stable ASMI and high‐stable eGDR; Group 2, high‐decrease ASMI and low‐decrease eGDR; Group 3, moderate‐stable ASMI and high‐decrease eGDR; Group 4, moderate‐decrease ASMI and moderate‐stable eGDR; Group 5, low‐decrease ASMI and low‐decrease eGDR. Dots show group‐specific mean observed levels while solid lines represent the best fitted trajectories. ASMI and eGDR were modelled as a function of follow‐up time. ASMI indicates appendicular skeletal muscle mass index; eGDR, estimated glucose disposal rate.
**Figure S5.** Stratified analysis of the association between baseline ASMI and eGDR and AF risk. (A) The association between baseline categorical measurement and AF risk. (B) The association between one‐SD increment of baseline ASMI and AF risk. (C) The association between one‐SD increment of baseline eGDR and AF risk. Models were adjusted for the covariates including age, sex, smoking status, alcohol consumption, exercise, SBP, DBP, heart rate, plasma lipids, FPG, history of dyslipidaemia and medications for antihypertension and antidyslipidaemia, COPD, eGFR, haemoglobin and CCA‐IMT and plaque. ASMI cut‐off was 7.0 kg/m^2^ for men and 5.7 kg/m^2^ for women. ASMI indicates appendicular skeletal muscle mass index; BMI, body mass index; eGFR, estimated glomerular filtration rate.
**Figure S6.** Direct effect of ASMI and indirect effect of eGDR on AF risk using mediation analysis. Models were adjusted for the covariates including age, sex, smoking status, alcohol consumption, exercise, SBP, DBP, heart rate, plasma lipids, FPG, history of dyslipidaemia and medications for antihypertension and antidyslipidaemia, COPD, eGFR, haemoglobin and CCA‐IMT and plaque. ASMI indicates appendicular skeletal muscle mass index; eGDR, estimated glucose disposal rate.

## Data Availability

The datasets supporting the conclusions presented in this work are available in the article or Supporting Information. Further inquiries can be directed to the corresponding authors.
