# Supplementary material for: Sarcopenia and Insulin Resistance Collective Effect on Atrial Fibrillation Risk: A Non‐Diabetic Elderly Cohort Study
Source: J Cachexia Sarcopenia Muscle. 2025 Feb 17;16(1):e13736. doi: 10.1002/jcsm.13736 (PMC11831525; doi:10.1002/jcsm.13736)
Supplement: Supplementary file 1 — Table S1. Association between baseline ASMI and the incidence of AF. Table S2. Different cumulative hazard of AF between the low and normal ASMI groups. Table S3. Interaction between baseline ASMI and eGDR on the incidence of AF. Table S4. Model fitting parameters for dual trajectories of ASMI and eGDR. Table S5. Association between dual trajectory of ASMI and eGDR and the incidence of AF. Table S6. Stratified analysis of the association between the collective effect of baseline ASMI and eGDR and AF risk. Table S7. Demographic and baseline characteristics of participants excluded from analysis of dual trajectory of ASMI and eGDR (n = 409). Figure S1. Cumulative hazard of AF between the low and normal ASMI groups. The cumulative hazard of overall AF in total participants (A), male (B) and female (C); the cumulative hazard of paroxysmal AF in total participants (D), male (E) and female (F); and the cumulative hazard of persistent AF in total participants (G), male (H) and female (I). ASMI indicates appendicular skeletal muscle mass index. Figure S2. Dose–response association of baseline ASMI and eGDR with paroxysmal and persistent AF risk analysed using restricted cubic splines model. The association between continuous measurement ASMI and paroxysmal AF risk in total participants (A), male (B) and female (C). The association between continuous measurement ASMI and persistent AF risk in total participants (D), male (E) and female (F). The association between continuous measurement eGDR and paroxysmal AF risk in total participants (G), male (H) and female (I). The association between continuous measurement eGDR and persistent AF risk in total participants (J), male (K) and female (L). ASMI indicates appendicular skeletal muscle mass index; eGDR, estimated glucose disposal rate. Figure S3. Cumulative hazard of paroxysmal and persistent AF in the groups classified by the dual trajectory of ASMI and eGDR. Cumulative hazard of paroxysmal AF in total participants (A), male ( [file JCSM-16-e13736-s001.docx]

**Sarcopenia and insulin resistance collective effect on atrial fibrillation risk: A non-diabetic elderly cohort study**

**Running title: Sarcopenia, insulin resistance, and atrial fibrillation**

Weike Liu^1^, Xin Wang^2^, Yuqi Guo^3,4^, Yumei Gao^5^, Huajing Song^3^, Yanli Yao^3,4^, Hua Zhang^3,4^, Zhendong Liu^3,4*^, Juan Wang^2*^

***Covariates***

Age, smoking status (current smoker or having smoked 100 or more cigarettes in the past, yes or no), alcohol consumption (drinking at least once a week or having consumed alcohol at least 6 months ago, yes or no), physical activity (engaging in physical exercise more than three times a week for at least 30 minutes each time, yes or no), history of hypertension and dyslipidemia (yes or no), medication use for antihypertension and anti-dyslipidemia (yes or no) were acquired using self-report questionnaires. Chronic obstructive pulmonary disease (COPD) was identified according to ICD-10 code J44 based on the review of medical records. Blood pressure was measured using an automatic digital BP monitor (HEM-7071, OMRON Healthcare Co, Ltd, Kyoto, Japan). FPG, triglycerides, total cholesterol (TCHO), low-density lipoprotein cholesterol, and high-density lipoprotein cholesterol plasma concentrations were determined by an enzymatic method using the Hitachi 7600 automated biochemical analyzer (Hitachi, Ltd, Tokyo, Japan). eGFR was evaluated using serum creatinine as described by the Chronic Kidney Disease Epidemiology Collaboration according to age, sex, and ethnicity [1,2]. Common carotid artery intima-media thickness (CCA-IMT) and plaque were measured using high-resolution ultrasound (Vivid *i*, GE Medical Systems Ultrasound Israel Ltd.). Plaque was defined as a focal structure encroaching into carotid arterial lumen of ≥0.5 mm or 50% of the surrounding IMT value, or a thickness of IMT >1.5 mm [3,4]. In addition, hemoglobin levels were included as covariates in this study. Anemia was defined as hemoglobin concentration less than 130 g/L for men and 120 g/L for women [5].

**Reference:**

1. Zhang Q, Guo Y, Li M, Yang R, Yao Y, Zhao Y, et al. Excessive dietary sodium intake augments long-term risk of atrial fibrillation in older adults with hyperglycemia: A community-based prospective cohort study. *Diabetes Metab.* 2023;49:101475.
2. Wei F, Sun X, Zhao Y, Zhang H, Diao Y, Liu Z. Excessive visit-to-visit glycemic variability independently deteriorates the progression of endothelial and renal dysfunction in patients with type 2 diabetes mellitus. *BMC Nephrol.* 2016;17:67.
3. Liu ZD, Wang L, Lu FH, Pan H, Zhao YX, Wang SJ, et al. Increased Th17 cell frequency concomitant with decreased Foxp3+ Treg cell frequency in the peripheral circulation of patients with carotid artery plaques. *Inflamm Res.* 2012;61:1155–1165.
4. Hou L, Wang X, Li P, Zhang H, Yao Y, Liu Z, et al. Adiposity modifies the association between heart failure risk and glucose metabolic disorder in older individuals: a community-based prospective cohort study. *Cardiovasc Diabetol*. 2024;23(1):318.
5. Pasricha SR, Rogers L, Branca F, Garcia-Casal MN. Measuring haemoglobin concentration to define anaemia: WHO guidelines. *Lancet.* 2024;403:1963–1966.

***Dual-trajectories of ASMI and eGDR in male and female:***

For male, group 1 characterized with high-slight-decrease ASMI and moderate-stable eGDR; Group2, moderate-decrease ASMI and moderate-stable eGDR; Group3, moderate-stable ASMI and moderate-stable eGDR; Group 4, high-significant-decrease ASMI and low-stable eGDR; Group 5, low-decrease ASMI and low-decrease eGDR. For female, group 1 characterized with high-stable ASMI and high-stable eGDR; Group 2, high-decrease ASMI and low-decrease eGDR; Group 3, moderate-stable ASMI and high-decrease eGDR; Group 4, moderate-decrease ASMI and moderate-stable eGDR; Group 5, low-decrease ASMI and low-decrease eGDR. The dual-trajectories of ASMI and eGDR presented as declined ASMI and eGDR were associated with high risk of overall, paroxysmal, and persistent AF in male and female (Figure 4B and C, Supplementary Figure 3B, C, E and F, and Supplemetary Table 4 and 5).

**Supplementary Tables:**

**Supplementary Table 1. Association between baseline ASMI and the incidence of AF**

|  | One-SD increment of baseline ASMI | | |  | One-SD increment of baseline ASMI | | |
| --- | --- | --- | --- | --- | --- | --- | --- |
|  | Total participant | Male | Female |  | Total participant | Male | Female |
| Overall AF |  |  |  |  |  |  |  |
| Unadjusted HR (95% CI) | 0.794 (0.741–0.850) | 0.830 (0.748–0.921) | 0.670 (0.606–0.742) |  | 0.350 (0.305–0.402) | 0.734 (0.671–0.803) | 0.609 (0.539–0.687) |
| Adjusted HR (95% CI) | 0.820 (0.768–0.875) | 0.853 (0.769–0.945) | 0.753 (0.676–0.839) |  | 0.637 (0.586–0.692) | 0.751 (0.687–0.822) | 0.635 (0.556–0.724) |
| Paroxysmal AF |  |  |  |  |  |  |  |
| Unadjusted HR (95% CI) | 0.802 (0.707–0.911) | 0.860 (0.780–0.948) | 0.712 (0.591–0.858) |  | 0.321 (0.249–0.414) | 0.731 (0.619–0.863) | 0.691 (0.552–0.865) |
| Adjusted HR (95% CI) | 0.821 (0.729–0.925) | 0.878 (0.796–0.969) | 0.781 (0.638–0.957) |  | 0.684 (0.591–0.792) | 0.738 (0.624–0.872) | 0.702 (0.568–0.867) |
| Persistent AF |  |  |  |  |  |  |  |
| Unadjusted HR (95% CI) | 0.787 (0.725–0.854) | 0.818 (0.724–0.925) | 0.654 (0.580–0.738) |  | 0.363 (0.308–0.427) | 0.736 (0.661–0.619) | 0.571 (0.493–0.662) |
| Adjusted HR (95% CI) | 0.820 (0.760–0.885) | 0.843 (0.747–0.952) | 0.743 (0.653–0.844) |  | 0.617 (0.559–0.681) | 0.758 (0.681–0.844) | 0.608 (0.519–0.713) |

Models were adjusted for the covariates including age, sex (for total participants models), smoking status, alcohol consumption, exercise, SBP, DBP, heart rate, plasma lipids, FPG, history of dyslipidemia and medications for antihypertension and anti-dyslipidemia, COPD, eGFR, hemoglobin, and CCA-IMT and plaque. ASMI indicates appendicular skeletal muscle mass index; HR,hazard ratio; CI, confidence interval.

**Supplementary Table 2. Different cumulative hazard of AF between the low and normal ASMI groups**

|  | Total participants | |  | Male | |  | Female | |
| --- | --- | --- | --- | --- | --- | --- | --- | --- |
|  | Normal ASMI | Low ASMI |  | Normal ASMI | Low ASMI |  | Normal ASMI | Low ASMI |
| Overall AF |  |  |  |  |  |  |  |  |
| Event/total | 587/6321 | 301/1739 |  | 318/2857 | 186/1157 |  | 269/3464 | 115/582 |
| Incidence rate (95% CI)* | 9.77 (9.03–10.50) | 18.73 (16.89–20.56) |  | 11.78 (10.60–12.97) | 17.36 (15.18–19.54) |  | 8.12 (7.21–9.03) | 21.46 (18.13–24.80) |
| Unadjusted HR (95% CI) | Reference | 1.962 (1.708–2.255) |  | Reference | 1.503 (1.255–1.802) |  | Reference | 2.715 (2.182–3.378) |
| Adjusted HR (95% CI) | Reference | 1.762 (1.528–2.032) |  | Reference | 1.418 (1.182–1.700) |  | Reference | 2.561 (2.049–3.202) |
| Paroxysmal AF |  |  |  |  |  |  |  |  |
| Event/total | 166/6321 | 88/1739 |  | 87/2857 | 54/1157 |  | 79/3464 | 34/582 |
| Incidence rate (95% CI)* | 2.76 (2.36–3.17) | 5.48 (4.41–6.54) |  | 3.22 (2.58–3.87) | 5.04 (3.78–6.30) |  | 2.39 (1.88–2.89) | 6.35 (4.36–0.83) |
| Unadjusted HR (95% CI) | Reference | 2.100 (1.624–2.716) |  | Reference | 1.594 (1.135–2.238) |  | Reference | 2.740 (1.833–4.097) |
| Adjusted HR (95% CI) | Reference | 1.897 (1.454–2.475) |  | Reference | 1.536 (1.092–2.161) |  | Reference | 2.712 (1.797–4.094) |
| Persistent AF |  |  |  |  |  |  |  |  |
| Event/total | 421/6321 | 213/1739 |  | 231/2857 | 132/1157 |  | 190/3464 | 81/582 |
| Incidence rate (95% CI)* | 7.00 (6.38–7.63) | 13.25 (11.66–14.85) |  | 8.56 (7.53–9.59) | 12.32 (10.43–14.21) |  | 5.74 (4.96–6.51) | 15.12 (12.21–18.03) |
| Unadjusted HR (95% CI) | Reference | 1.936 (1.642–2.283) |  | Reference | 1.469 (1.186–1.820) |  | Reference | 2.705 (2.085–3.509) |
| Adjusted HR (95% CI) | Reference | 1.710 (1.444–2.025) |  | Reference | 1.374 (1.109–1.703) |  | Reference | 2.499 (1.916–3.259) |

*per 1000 person-years

Models were adjusted for the covariates including age, sex (for total participants models), smoking status, alcohol consumption, exercise, SBP, DBP, heart rate, plasma lipids, FPG, history of dyslipidemia and medications for antihypertension and anti-dyslipidemia, COPD, eGFR, hemoglobin, and CCA-IMT and plaque. ASMI indicates appendicular skeletal muscle mass index; HR,hazard ratio; CI, confidence interval.

**Supplementary Table 3. Interaction between baseline ASMI and eGDR on the incidence of AF**

|  |  | Interaction between low ASMI group and eGDR group | | |  | Interaction between one-SD ASMI and eGDR increments |
| --- | --- | --- | --- | --- | --- | --- |
|  |  | High eGDR | Moderate eGDR | Low eGDR |  |  |
| Overall AF |  |  |  |  |  |  |
|  | Total participants |  |  |  |  |  |
|  | Unadjusted HR (95% CI) | Reference | 1.295 (1.142–1.470) | 1.912 (1.729–2.114) |  | 0.880 (0.822–0.941) |
|  | Adjusted HR (95% CI) | Reference | 1.290 (1.136–1.464) | 1.666 (1.492–1.861) |  | 0.896 (0.839–0.957) |
|  | Male |  |  |  |  |  |
|  | Unadjusted HR (95% CI) | Reference | 1.172 (1.027–1.337) | 1.656 (1.458–1.880) |  | 0.904 (0.841–0.972) |
|  | Adjusted HR (95% CI) | Reference | 1.158 (1.009–1.329) | 1.444 (1.256–1.659) |  | 0.911 (0.843–0.984) |
|  | Female |  |  |  |  |  |
|  | Unadjusted HR (95% CI) | Reference | 1.573 (1.294–1.912) | 2.295 (1.944–2.709) |  | 0.842 (0.764–0.929) |
|  | Adjusted HR (95% CI) | Reference | 1.537 (1.262–1.870) | 2.150 (1.795–2.575) |  | 0.845 (0.767–0.932) |
| Paroxysmal AF |  |  |  |  |  |  |
|  | Total participants |  |  |  |  |  |
|  | Unadjusted HR (95% CI) | Reference | 1.416 (1.133–1.770) | 1.698 (1.397–2.065) |  | 0.943 (0.925–0.961) |
|  | Adjusted HR (95% CI) | Reference | 1.404 (1.119–1.760) | 1.583 (1.281–1.957) |  | 0.908 (0.856–0.963) |
|  | Male |  |  |  |  |  |
|  | Unadjusted HR (95% CI) | Reference | 1.150 (1.008–1.312) | 1.497 (1.171–1.913) |  | 0.937 (0.883–0.994) |
|  | Adjusted HR (95% CI) | Reference | 1.145 (1.001–1.309) | 1.319 (1.006-1.729) |  | 0.945 (0.894–0.999) |
|  | Female |  |  |  |  |  |
|  | Unadjusted HR (95% CI) | Reference | 1.852 (1.323–2.581) | 1.974 (1.422–2.740) |  | 0.895 (0.824–0.972) |
|  | Adjusted HR (95% CI) | Reference | 1.819 (1.295–2.554) | 1.945 (1.364–2.772) |  | 0.904 (0.834–0.980) |
| Persistent AF |  |  |  |  |  |  |
|  | Total participants |  |  |  |  |  |
|  | Unadjusted HR (95% CI) | Reference | 1.256 (1.076–1.466) | 1.995 (1.774–2.243) |  | 0.839 (0.744–0.946) |
|  | Adjusted HR (95% CI) | Reference | 1.241 (1.064–1.448) | 1.791 (1.578–2.032) |  | 0.883 (0.820–0.951) |
|  | Male |  |  |  |  |  |
|  | Unadjusted HR (95% CI) | Reference | 1.158 (1.048–1.280) | 1.719 (1.481–1.995) |  | 0.900 (0.813–0.995) |
|  | Adjusted HR (95% CI) | Reference | 1.112 (1.011–1.223) | 1.491 (1.268–1.752) |  | 0.902 (0.814–0.998) |
|  | Female |  |  |  |  |  |
|  | Unadjusted HR (95% CI) | Reference | 1.446 (1.136–1.839) | 2.415 (1.991–2.928) |  | 0.823 (0.734–0.923) |
|  | Adjusted HR (95% CI) | Reference | 1.414 (1.110–1.801) | 2.216 (1.797–2.734) |  | 0.825 (0.734–0.927) |

Models were adjusted for the covariates including age, sex, smoking status, alcohol consumption, exercise, SBP, DBP, heart rate, plasma lipids, FPG, history of dyslipidemia and medications for antihypertension and anti-dyslipidemia, COPD, eGFR, hemoglobin, and CCA-IMT and plaque. ASMI indicates appendicular skeletal muscle mass index; eGFR, estimated glomerular filtration rate; HR,hazard ratio; CI, confidence interval.

**Supplementary Table 4. Model fitting parameters for dual-trajectories of ASMI and eGDR**

|  | Classification | | | | | |
| --- | --- | --- | --- | --- | --- | --- |
| Latent class | 1 | 2 | 3 | 4 | 5 | 6 |
| Total participants |  |  |  |  |  |  |
| BIC | 80868.54 | 80834.03 | 80797.73 | 80727.67 | 78868.31 | 80691.90 |
| AIC | 80793.71 | 80785.26 | 80704.48 | 80683.92 | 78817.53 | 80622.17 |
| APP | - | 0.91/0.87 | 0.85/0.82/0.84 | 0.83/0.82/0.81/0.81 | 0.81/0.80/0.79/0.80/0.78 | 0.79/0.76/0.81/0.82/0.72/0.76 |
| Log-likelihood | -42927.74 | -42361.24 | -41300.76 | -40336.51 | -39353.67 | -39300.16 |
| Entropy | - | 0.874 | 0.836 | 0.819 | 0.883 | 0.808 |
| GM (%) | 7651 (100.00) | 3759/3892  (49.13/50.87) | 2475/1955/3221  (32.35/25.55/42.10) | 1874/1790/1612/2375  (24.49/23.40/21.07/31.04) | 1630/1657/1127/1351/1886  (21.30/21.66/14.73/17.66/24.65) | 1566/1793/1376/1778/292/846  (20.47/23.43/17.98/23.24/3.82/11.06) |
| Male |  |  |  |  |  |  |
| BIC | 78694.62 | 78458.38 | 77051.67 | 76996.72 | 75106.29 | 75979.23 |
| AIC | 78547.65 | 78248.90 | 77042.15 | 76896.54 | 74138.62 | 75236.69 |
| APP | - | 0.93/0.86 | 0.87/0.85/0.85 | 0.84/0.84/0.81/0.82 | 0.83/0.82/0.80/0.81/0.79 | 0.81/0.79/0.80/0.78/0.76/0.78 |
| Log-likelihood | -43154.21 | -42886.57 | -42539.55 | -40984.26 | -40152.39 | -39827.43 |
| Entropy | - | 0.867 | 0.854 | 0.861 | 0.877 | 0.832 |
| GM (%) | 3816  (100.00) | 1804/2012  (47.27/52.73) | 836/1624/1356  (21.91/42.56/35.53) | 667/1198/995/956  (17.48/31.39/26.08/25.05) | 431/1031/841/881/632  (11.29/27.02/22.04/23.09/16.56) | 362/172/996/925/633/728  (9.49/4.51/26.10/24.23/16.59/19.08) |
| Female |  |  |  |  |  |  |
| BIC | 82671.49 | 82439.50 | 81636.62 | 81017.94 | 80061.38 | 80163.49 |
| AIC | 82196.91 | 81945.29 | 81428.55 | 80843.34 | 79271.18 | 79435.32 |
| APP | - | 0.90/0.88 | 0.87/0.84/0.85 | 0.86/0.84/0.83 | 0.84/0.81/0.78/0.81/0.79 | 0.82/0.79/0.80/0.76/0.82/0.77 |
| Log-likelihood | -40381.81 | -40105.74 | -39964.52 | -39883.33 | -38239.09 | -38216.57 |
| Entropy | - | 0.875 | 0.842 | 0.836 | 0.869 | 0.843 |
| GM (%) | 3835 (100.00) | 1873/1962  (48.84/51.16) | 819/1363/1653  (21.36/35.54/43.10) | 704/1396/1114/621  (18.36/36.40/29.05/16.19) | 596/588/1280/884/487  (15.54/15.33/33.38/23.05/12.70) | 180/559/1227/842/184/843  (4.69/14.58/31.99/21.96/4.80/21.98) |

Abbreviations: AIC, Akaike information criterion; BIC, Bayesian information criterion; APP, average posterior probability; GM, group membership.

**Supplementary Table 5. Association between dual-trajectory of ASMI and eGDR and the incidence of AF**

|  | Dual-trajectory of ASMI and eGDR levels | | | | |
| --- | --- | --- | --- | --- | --- |
|  | Group 1 | Group 2 | Group 3 | Group 4 | Group 5 |
| Dual-trajectory of ASMI and eGDR levels in total participants | | | | | |
| Overall |  |  |  |  |  |
| Event/total | 246/1630 | 166/1657 | 128/1127 | 91/1351 | 235/1886 |
| Incidence rate (95% CI)* | 16.05 (14.27–17.83) | 10.55 (9.07–12.03) | 12.02 (10.12–13.92) | 7.03 (5.67–8.39) | 13.29 (11.76–14.82) |
| Unadjusted HR (95% CI) | 2.636 (2.039–3.407) | 1.546 (1.196–1.997) | 1.876 (1.420–2.479) | Reference | 1.925 (1.511–2.452) |
| Adjusted HR (95% CI) | 2.255 (1.769–2.876) | 1.513 (1.171–1.955) | 1.630 (1.244–2.136) | Reference | 1.893 (1.491–2.403) |
| Paroxysmal AF |  |  |  |  |  |
| Event/total | 65/1630 | 50/1657 | 36/1127 | 29/1351 | 66/1886 |
| Incidence rate (95% CI)* | 4.24 (3.26–5.22) | 3.18 (2.33–4.02) | 3.38 (2.32–4.43) | 2.24 (1.45–3.03) | 3.73 (2.88–4.59) |
| Unadjusted HR (95% CI) | 1.991 (1.235–3.208) | 1.441 (0.911–2.281) | 1.563 (0.934–2.615) | Reference | 1.889 (0.986–3.619) |
| Adjusted HR (95% CI) | 1.921 (1.234–2.991) | 1.432 (0.906–2.263) | 1.489 (0.910–2.435) | Reference | 1.494 (0.961–2.323) |
| Persistent AF |  |  |  |  |  |
| Event/total | 181/1630 | 116/1657 | 92/1127 | 62/1351 | 169/1886 |
| Incidence rate (95% CI)* | 11.81 (10.24–13.38) | 7.37 (6.11–8.63) | 8.64 (7.00–10.28) | 4.79 (3.65–5.93) | 9.56 (8.23–10.88) |
| Unadjusted HR (95% CI) | 2.939 (2.165–3.990) | 1.597 (1.173–2.176) | 2.015 (1.445–2.809) | Reference | 2.416 (1.626–3.590) |
| Adjusted HR (95% CI) | 2.494 (1.869–3.328) | 1.554 (1.142–2.116) | 1.801 (1.305–2.486) | Reference | 2.031 (1.518–2.717) |
| Dual-trajectory of ASMI and eGDR levels in male | | | | | |
| Overall |  |  |  |  |  |
| Event/total | 42/431 | 113/1031 | 75/841 | 149/881 | 111/632 |
| Incidence rate (95% CI)* | 10.23 (7.37–13.09) | 11.61 (9.65–13.56) | 9.34 (7.37–11.31) | 18.25 (15.70–20.80) | 18.97 (15.91–22.02) |
| Unadjusted HR (95% CI) | 1.175 (0.803–1.719) | 1.328 (0.980–1.798) | Reference | 2.000 (1.515–2.640) | 2.081 (1.553–2.790) |
| Adjusted HR (95% CI) | 1.088 (0.745–1.587) | 1.266 (0.946–1.695) | Reference | 1.654 (1.235–2.217) | 1.859 (1.374–2.516) |
| Paroxysmal AF |  |  |  |  |  |
| Event/total | 9/431 | 38/1031 | 22/841 | 35/881 | 32/632 |
| Incidence rate (95% CI)* | 2.19 (0.81–3.57) | 3.90 (2.72–5.09) | 2.74 (1.64–3.84) | 4.29 (2.95–5.63) | 5.47 (3.70–7.24) |
| Unadjusted HR (95% CI) | 0.795 (0.366–1.728) | 1.451 (0.859–2.452) | Reference | 1.601 (0.939–2.729) | 2.045 (1.188–3.519) |
| Adjusted HR (95% CI) | 0.840 (0.385–1.834) | 1.432 (0.827–2.477) | Reference | 1.342 (0.763–2.358) | 1.865 (1.060–3.281) |
| Persistent AF |  |  |  |  |  |
| Event/total | 33/431 | 75/1031 | 53/841 | 114/881 | 79/632 |
| Incidence rate (95% CI)* | 8.03 (5.47–10.60) | 7.70 (6.08–9.33) | 6.60 (4.92–0.828) | 13.97 (11.68–16.26) | 13.50 (10.84–16.16) |
| Unadjusted HR (95% CI) | 1.316 (0.849–2.039) | 1.276 (0.886–1.837) | Reference | 2.166 (1.564–3.000) | 2.097 (1.480–2.969) |
| Adjusted HR (95% CI) | 1.209 (0.783–1.867) | 1.190 (0.837–1.691) | Reference | 1.872 (1.327–2.640) | 1.865 (1.303–2.670) |
| Dual-trajectory of ASMI and eGDR levels in female | | | | | |
| Overall |  |  |  |  |  |
| Event/total | 34/596 | 63/588 | 94/1280 | 72/884 | 113/487 |
| Incidence rate (95% CI)* | 5.93 (4.04–7.83) | 11.32 (8.76–13.88) | 7.65 (6.19–9.11) | 8.53 (6.69–10.37) | 25.50 (21.63–29.37) |
| Unadjusted HR (95% CI) | Reference | 1.975 (1.290–3.023) | 1.298 (0.877–1.922) | 1.446 (0.955–2.191) | 4.446 (3.030–6.524) |
| Adjusted HR (95% CI) | Reference | 1.961 (1.292–2.976) | 1.295 (0.875–1.917) | 1.433 (0.853–2.154) | 4.167 (2.785–6.235) |
| Paroxysmal AF |  |  |  |  |  |
| Event/total | 9/596 | 23/588 | 34/1280 | 19/884 | 25/487 |
| Incidence rate (95% CI)* | 1.57 (0.57–2.57) | 4.13 (2.52–5.74) | 2.77 (1.87–3.67) | 2.25 (1.27–3.23) | 5.64 (3.59–7.69) |
| Unadjusted HR (95% CI) | Reference | 2.799 (1.273–6.156) | 1.772 (0.850–3.695) | 1.444 (0.644–3.239) | 3.721 (1.737–7.973) |
| Adjusted HR (95% CI) | Reference | 2.719 (1.258–5.877) | 1.744 (0.836–3.639) | 1.426 (0.645–3.151) | 3.690 (1.662–8.193) |
| Persistent AF |  |  |  |  |  |
| Event/total | 25/596 | 40/588 | 60/1280 | 53/884 | 88/487 |
| Incidence rate (95% CI)* | 4.36 (2.72–6.00) | 7.19 (5.10–9.27) | 4.88 (3.70–6.06) | 6.28 (4.68–7.88) | 19.86 (16.31–23.40) |
| Unadjusted HR (95% CI) | Reference | 1.689 (1.025–2.785) | 1.136 (0.712–1.812) | 1.446 (0.891–2.347) | 4.707 (3.018–7.339) |
| Adjusted HR (95% CI) | Reference | 1.695 (1.018–2.823) | 1.124 (0.705–1.792) | 1.435 (0.892–2.309) | 4.279 (2.680–6.830) |

*per 1000 person-years

For dual-trajectory of ASMI and eGDR levels in total participants, Group 1 was high-decrease ASMI and high-decrease eGDR; group 2, high-decrease ASMI and moderate-stable eGDR; group 3, moderate-stable ASMI and moderate-decrease eGDR; group 4, moderate-stable ASMI and moderate-stable eGDR; group 5, high-decrease ASMI and low-stable eGDR. For dual-trajectory of ASMI and eGDR levels in male, Group 1 was high-slight-decrease ASMI and moderate-stable eGDR; Group2, moderate-decrease ASMI and moderate-stable eGDR; Group3, moderate-stable ASMI and moderate-stable eGDR; Group 4, high-significant-decrease ASMI and low-stable eGDR; Group 5, low-decrease ASMI and low-decrease eGDR. For dual-trajectory of ASMI and eGDR levels in female, Group 1 was high-stable ASMI and high-stable eGDR; Group 2, high-decrease ASMI and low-decrease eGDR; Group 3, moderate-stable ASMI and high-decrease eGDR; Group 4, moderate-decrease ASMI and moderate-stable eGDR; Group 5, low-decrease ASMI and low-decrease eGDR.

Models were adjusted for the covariates including age, sex, smoking status, alcohol consumption, exercise, SBP, DBP, heart rate, plasma lipids, FPG, history of dyslipidemia and medications for antihypertension and anti-dyslipidemia, COPD, eGFR, hemoglobin, and CCA-IMT and plaque. ASMI indicates appendicular skeletal muscle mass index; eGFR, estimated glomerular filtration rate; HR,hazard ratio; CI, confidence interval.

**Supplementary Table 6. Stratified analysis of the association between the collective effect of baseline ASMI and eGDR and AF risk**

| Subgroup | | Interaction between categorical measurements of baseline ASMI and eGDR with AF risk | |  | Interaction between one-SD increments of baseline ASMI and eGDR with AF risk | |
| --- | --- | --- | --- | --- | --- | --- |
|  |  | Unadjusted HR (95% CI) | Adjusted HR (95% CI) |  | Unadjusted HR (95% CI) | Adjusted HR (95% CI) |
| Age, years | | | | | | |
|  | <70 | 1.394 (1.300–1.494) | 1.286 (1.198–1.265) |  | 0.846 (0.781–0.917) | 0.868 (0.801–0.842) |
|  | ≥70 | 1.349 (1.223–1.487) | 1.238 (1.119–1.368) |  | 0.940 (0.836–1.054) | 0.951 (0.848–1.068) |
| BMI, kg/m^2^ | | | | | | |
|  | <28 | 1.371 (1.270–1.480) | 1.279 (1.181–1.385) |  | 0.881 (0.805–0.964) | 0.903 (0.826–0.987) |
|  | ≥28 | 1.380 (1.269–1.501) | 1.260 (1.156–1.373) |  | 0.876 (0.800–0.959) | 0.887 (0.805–0.977) |
| Regular physical activity | | | | | | |
|  | Yes | 1.323 (1.221–1.435) | 1.204 (1.108–1.307) |  | 0.910 (0.829–0.999) | 0.932 (0.846–1.027) |
|  | No | 1.453 (1.342–1.573) | 1.332 (1.227–1.446) |  | 0.852 (0.778–0.933) | 0.864 (0.791–0.944) |
| Anemia | | | | | | |
|  | Yes | 1.293 (1.204–1.390) | 1.205 (1.121–1.295) |  | 0.888 (0.816–0.967) | 0.876 (0.807–0.952) |
|  | No | 1.515 (1.382–1.661) | 1.382 (1.253–1.525) |  | 0.867 (0.796–0.943) | 0.900 (0.813–0.996) |
| eGFR, ml/min/1.73m^2^ | | | | | | |
|  | ≥90 | 1.448 (1.308–1.602) | 1.431 (1.284–1.594) |  | 0.958 (0.857–1.072) | 0.968 (0.870–1.076) |
|  | <90 | 1.240 (1.159–1.326) | 1.209 (1.128–1.295) |  | 0.832 (0.769–0.901) | 0.842 (0.777–0.912) |

Models were adjusted for the covariates including age, sex, smoking status, alcohol consumption, exercise, SBP, DBP, heart rate, plasma lipids, FPG, history of dyslipidemia and medications for antihypertension and anti-dyslipidemia, COPD, eGFR, hemoglobin, and CCA-IMT and plaque. ASMI indicates appendicular skeletal muscle mass index; eGFR, estimated glomerular filtration rate; HR,hazard ratio; CI, confidence interval.

**Supplementary Table 7. Demographic and baseline characteristics of participants excluded from analysis of dual-trajectory of ASMI and eGDR (*n*=409)**

|  | Value　(*n*=409) |
| --- | --- |
| Age, years | 67.98±4.77 |
| Sex, *n* (%) |  |
| Female | 211 (51.59) |
| Male | 198 (48.41) |
| Smoking, *n* (%) | 92 (22.49) |
| Alcohol consumption, *n* (%) | 122 (29.83) |
| Physical exercise, *n* (%) | 215 (52.57) |
| WC, cm | 92.86±6.72 |
| BMI, kg/m^2^ | 24.52±2.48 |
| SBP, mm Hg | 147.78±9.85 |
| DBP, mm Hg | 70.32±7.28 |
| Heart rate, beats/min | 72.84±8.37 |
| TCHO, mmol/L | 4.79±0.65 |
| Triglycerides, mmol/L | 1.55±0.49 |
| HDL-c, mmol/L | 1.27±0.37 |
| LDL-c, mmol/L | 2.93±0.53 |
| FPG, mmol/L | 5.45±0.80 |
| HbA1c, % | 5.15±0.36 |
| Hypertension, *n* (%) | 278 (67.97) |
| Dyslipidemia, *n* (%) | 183 (44.74) |
| Antihypertensive medication, *n* (%) | 219 (53.55) |
| Anti-dyslipidemia medication, *n* (%) | 63 (15.40) |
| COPD, n (%) | 34 (8.3) |
| Hemoglobin, g/L | 125.24±7.80 |
| Anemia, n (%) | 110 (26.89) |
| CCA-IMT, mm | 1.28±0.24 |
| CCA-plaque, n (%) | 78 (19.1) |
| eGFR, ml/min/1.73 m^2^ | 92.30 (88.03–95.77) |
| ASMI, kg/m^2^ | 7.17±1.04 |
| eGDR, mg/kg/min | 9.74±1.84 |

Results are mean±SD, median with interquartile range, or frequencies with percentages. ASMI indicates appendicular skeletal muscle mass index; eGDR, estimated glucose disposal rate; WC, waist circumference; BMI, body mass index; SBP, systolic blood pressure; DBP, diastolic blood pressure; TCHO, total cholesterol; HDL-c, high-density lipoprotein cholesterol; LDL-c, low-density lipoprotein cholesterol; FPG, fasting plasma glucose; COPD, chronic obstructive pulmonary disease; CCA-IMT, common carotid artery intima-media thickness; eGFR, estimated glomerular filtration rate.

**Supplementary Figures：**

**
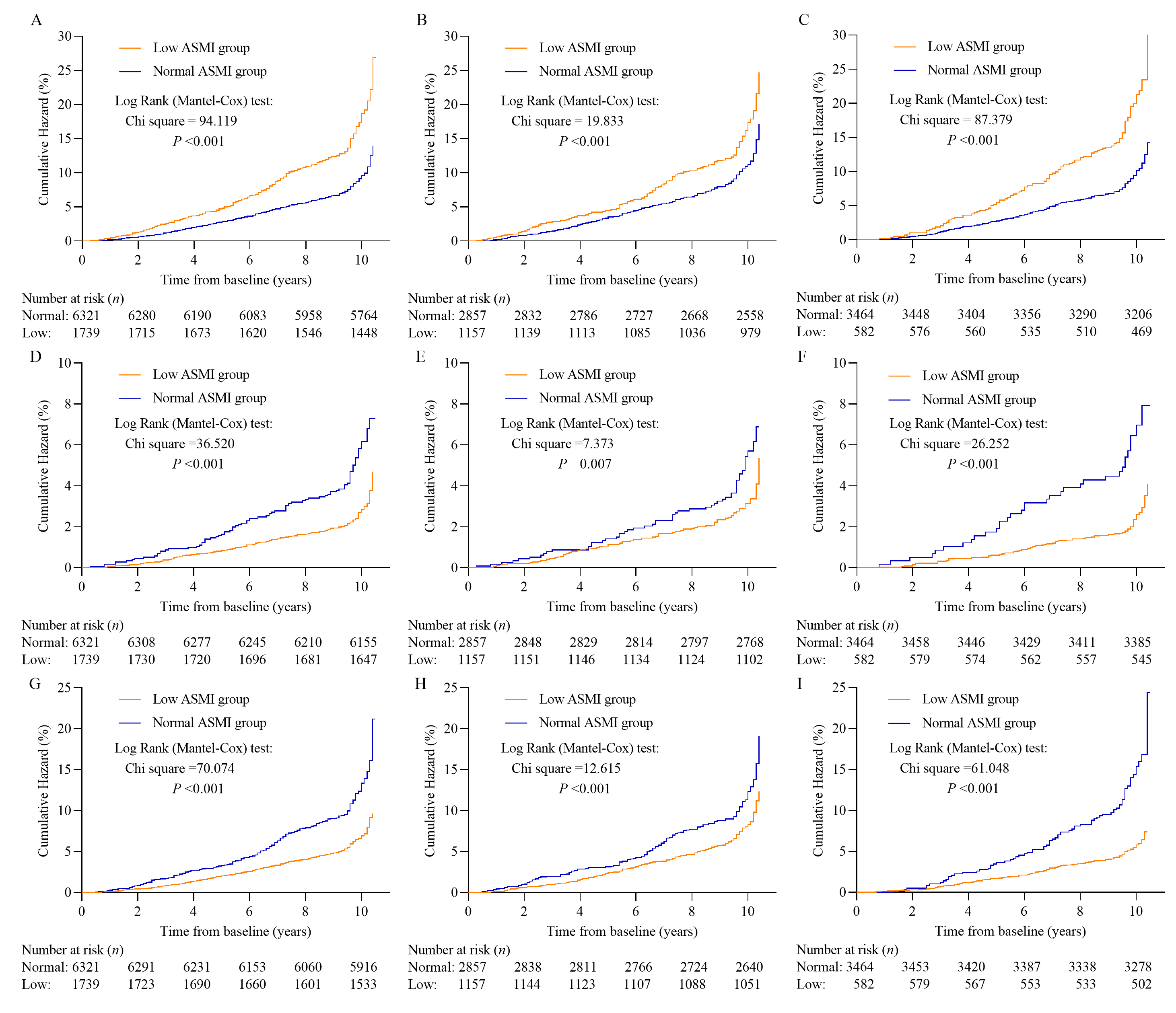
**

**Supplementary Figure 1. Cumulative hazard of AF between the low and normal ASMI groups.** The cumulative hazard of overall AF in total participants (A), male (B), and female (C); the cumulative hazard of paroxysmal AF in total participants (D), male (E), and female (F); and the cumulative hazard of persistent AF in total participants (G), male (H), and female (I). ASMI indicates appendicular skeletal muscle mass index.

**
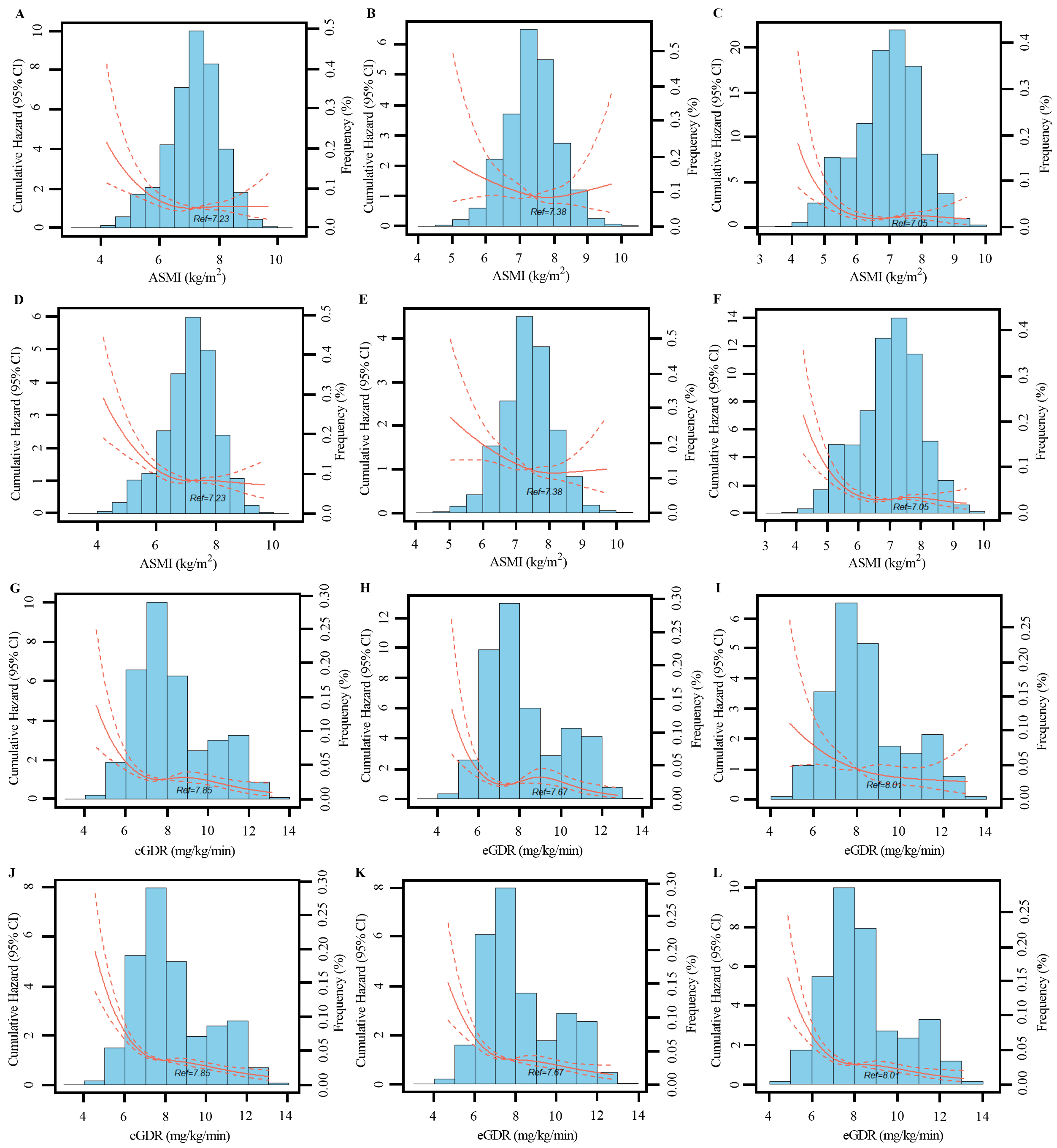
**

**Supplementary Figure 2. Dose-response association of baseline ASMI and eGDR with paroxysmal and persistent AF risk analyzed using restricted cubic splines model.** The association between continuous measurement ASMI and paroxysmal AF risk in total participants (A), male (B), and female (C). The association between continuous measurement ASMI and persistent AF risk in total participants (D), male (E), and female (F). The association between continuous measurement eGDR and paroxysmal AF risk in total participants (G), male (H), and female (I). The association between continuous measurement eGDR and persistent AF risk in total participants (J), male (K), and female (L). ASMI indicates appendicular skeletal muscle mass index; eGDR, and estimated glucose disposal rate.

**
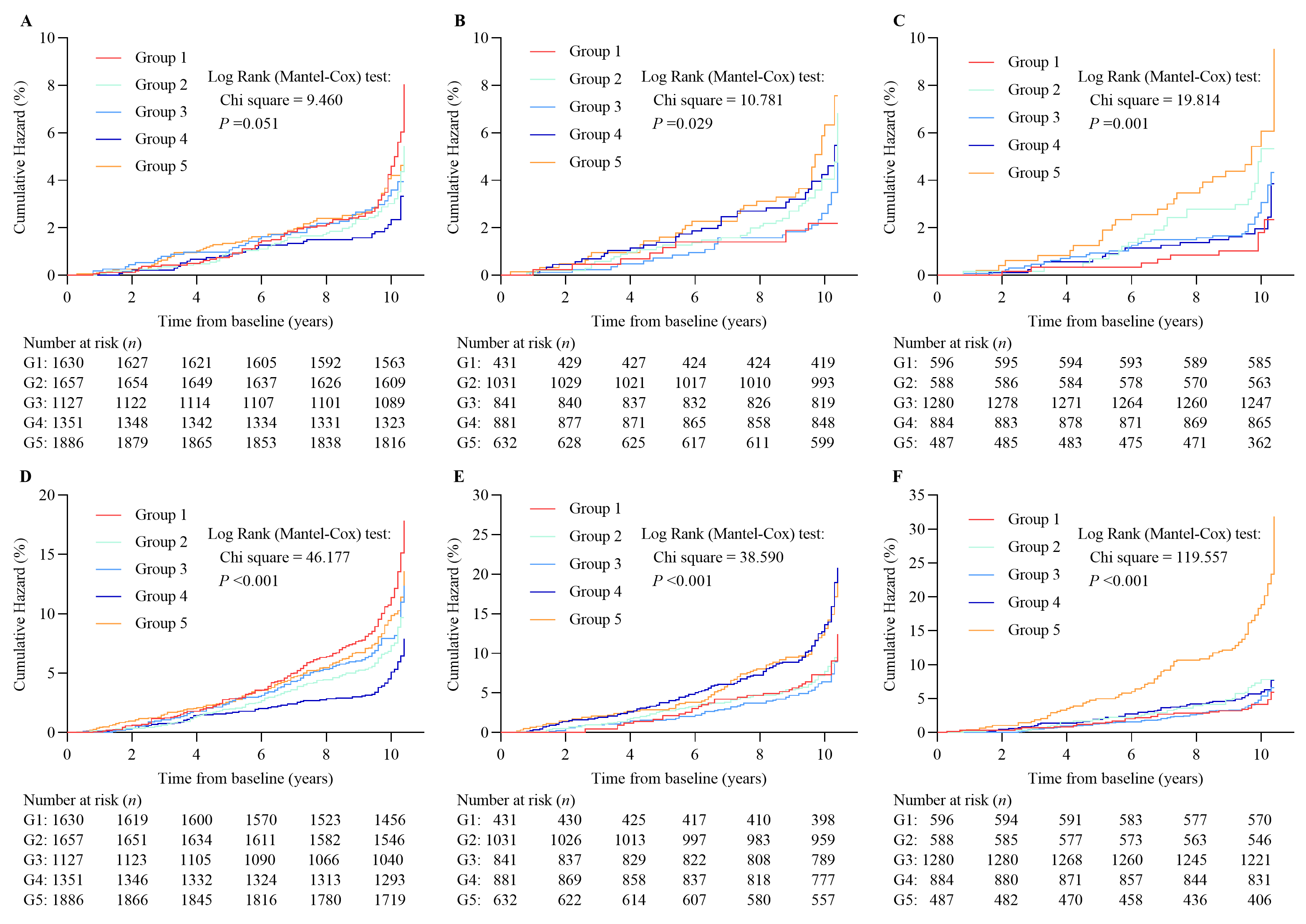
**

**Supplementary Figure 3. Cumulative hazard of paroxysmal and persistent AF in the groups classified by the dual-trajectory of ASMI and eGDR.** Cumulative hazard of paroxysmal AF in total participants (A), male (B), and female (C); cumulative hazard of persistent AF in total participants (D), male (E), and female (F). For total participants, Group 1 was high-decrease ASMI and high-decrease eGDR; group 2, high-decrease ASMI and moderate-stable eGDR; group 3, moderate-stable ASMI and moderate-decrease eGDR; group 4, moderate-stable ASMI and moderate-stable eGDR; group 5, high-decrease ASMI and low-stable eGDR. For male, Group 1 was high-slight-decrease ASMI and moderate-stable eGDR; Group2, moderate-decrease ASMI and moderate-stable eGDR; Group3, moderate-stable ASMI and moderate-stable eGDR; Group 4, high-significant-decrease ASMI and low-stable eGDR; Group 5, low-decrease ASMI and low-decrease eGDR. For female, Group 1 was high-stable ASMI and high-stable eGDR; Group 2, high-decrease ASMI and low-decrease eGDR; Group 3, moderate-stable ASMI and high-decrease eGDR; Group 4, moderate-decrease ASMI and moderate-stable eGDR; Group 5, low-decrease ASMI and low-decrease eGDR.

**
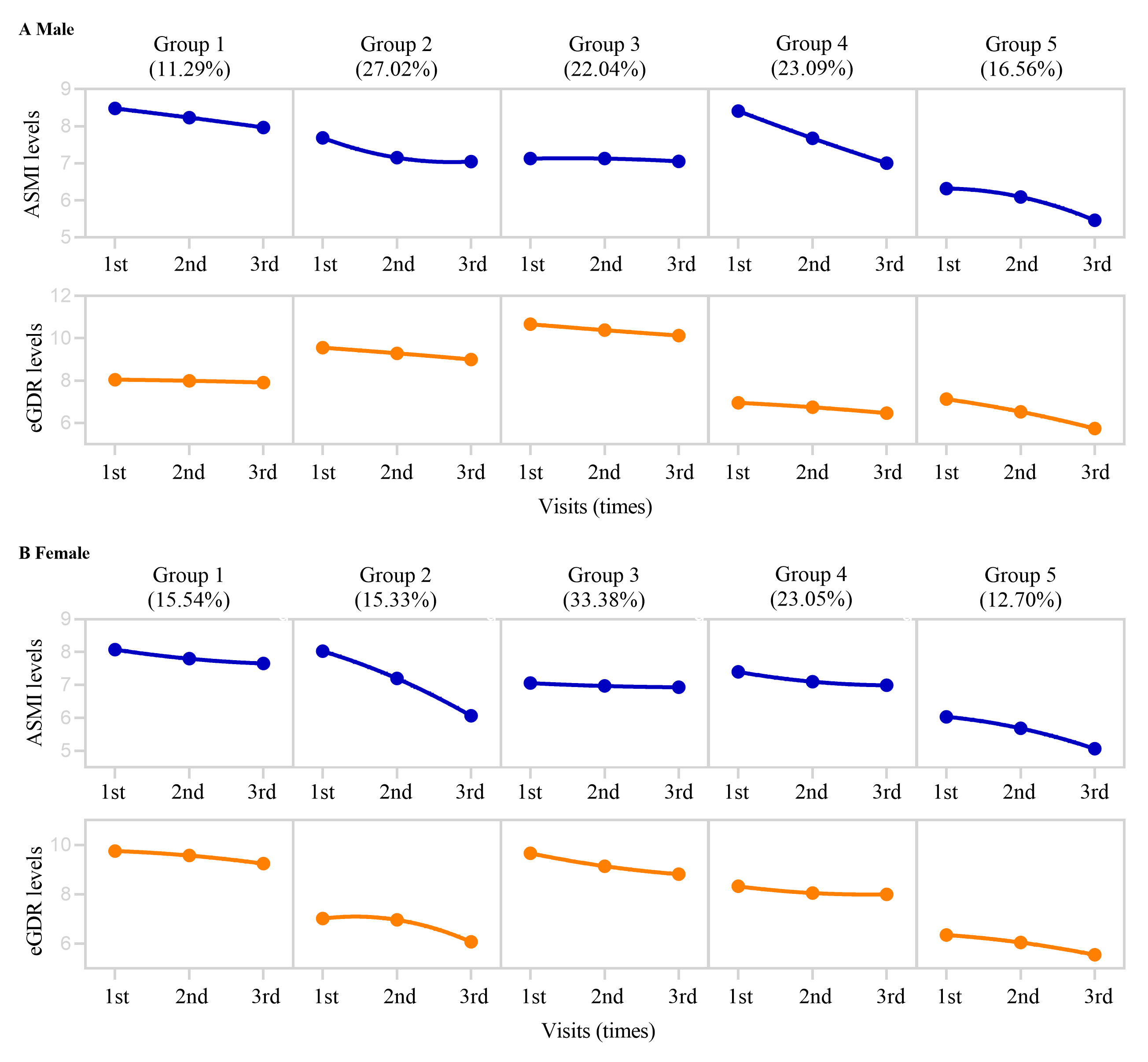
**

**Supplementary Figure 4. Dual-trajectories of ASMI and eGDR identified in male and female using group-based dual-trajectory modeling.** (A) Dual-trajectories of ASMI and eGDR in male. Group 1 was high-slight-decrease ASMI and moderate-stable eGDR; Group2, moderate-decrease ASMI and moderate-stable eGDR; Group3, moderate-stable ASMI and moderate-stable eGDR; Group 4, high-significant-decrease ASMI and low-stable eGDR; Group 5, low-decrease ASMI and low-decrease eGDR. (B) Dual-trajectories of ASMI and eGDR in female. Group 1 was high-stable ASMI and high-stable eGDR; Group 2, high-decrease ASMI and low-decrease eGDR; Group 3, moderate-stable ASMI and high-decrease eGDR; Group 4, moderate-decrease ASMI and moderate-stable eGDR; Group 5, low-decrease ASMI and low-decrease eGDR. Dots show group-specific mean observed levels while solid lines represent the best fitted trajectories. ASMI and eGDR were modeled as a function of follow-up time. ASMI indicates appendicular skeletal muscle mass index; eGDR, and estimated glucose disposal rate.

**
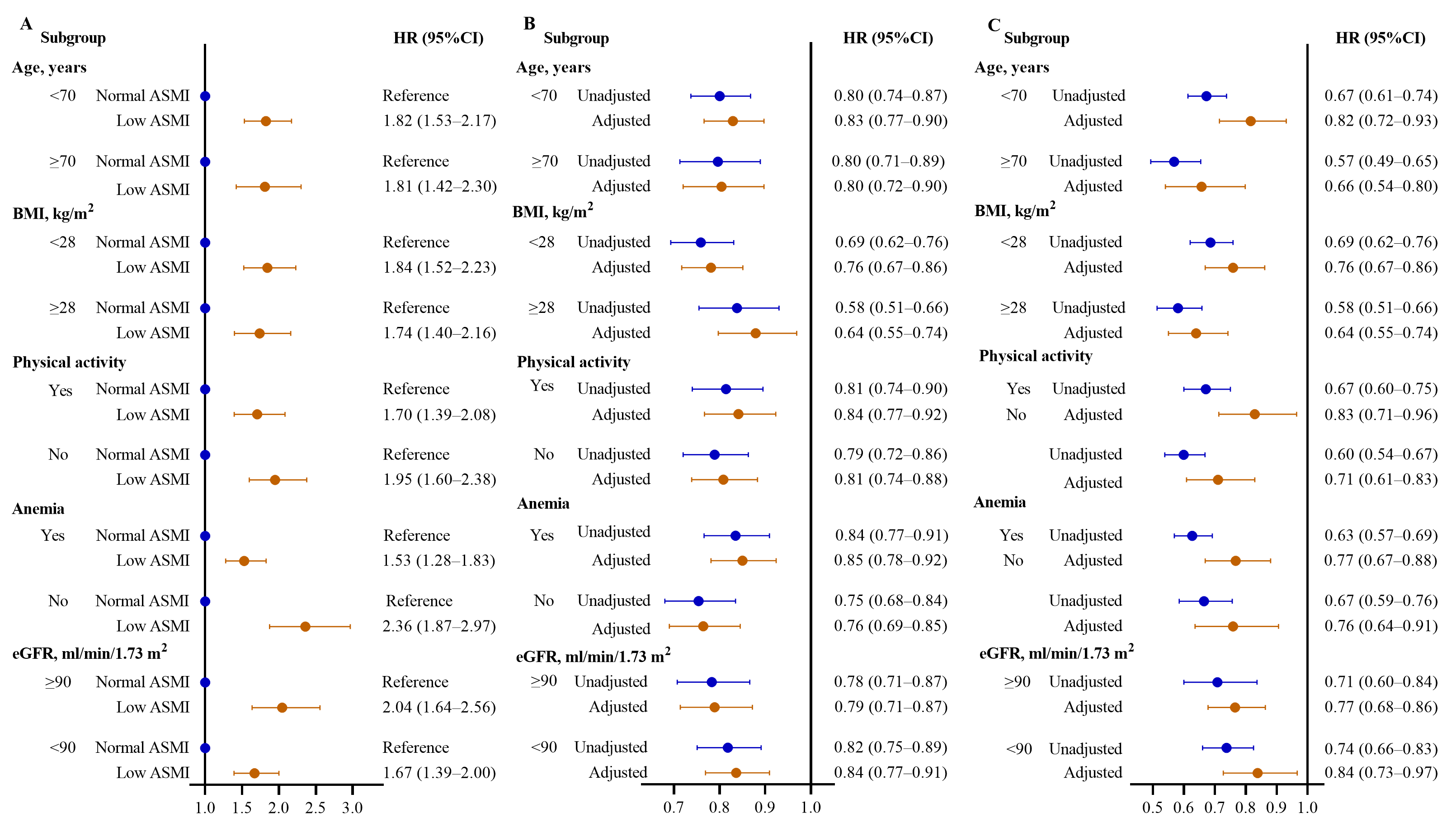
**

**Supplementary Figure 5. Stratified analysis of the association between baseline ASMI and eGDR and AF risk.** (A) The association between baseline categorical measurement and AF risk. (B) The association between one-SD increment of baseline ASMI and AF risk. (C) The association between one-SD increment of baseline eGDR and AF risk. Models were adjusted for the covariates including age, sex, smoking status, alcohol consumption, exercise, SBP, DBP, heart rate, plasma lipids, FPG, history of dyslipidemia and medications for antihypertension and anti-dyslipidemia, COPD, eGFR, hemoglobin, and CCA-IMT and plaque. ASMI cut-off was 7.0 kg/m^2^ for men and 5.7 kg/m^2^ for women. ASMI indicates appendicular skeletal muscle mass index; BMI, body mass index; eGFR, estimated glomerular filtration rate.

**
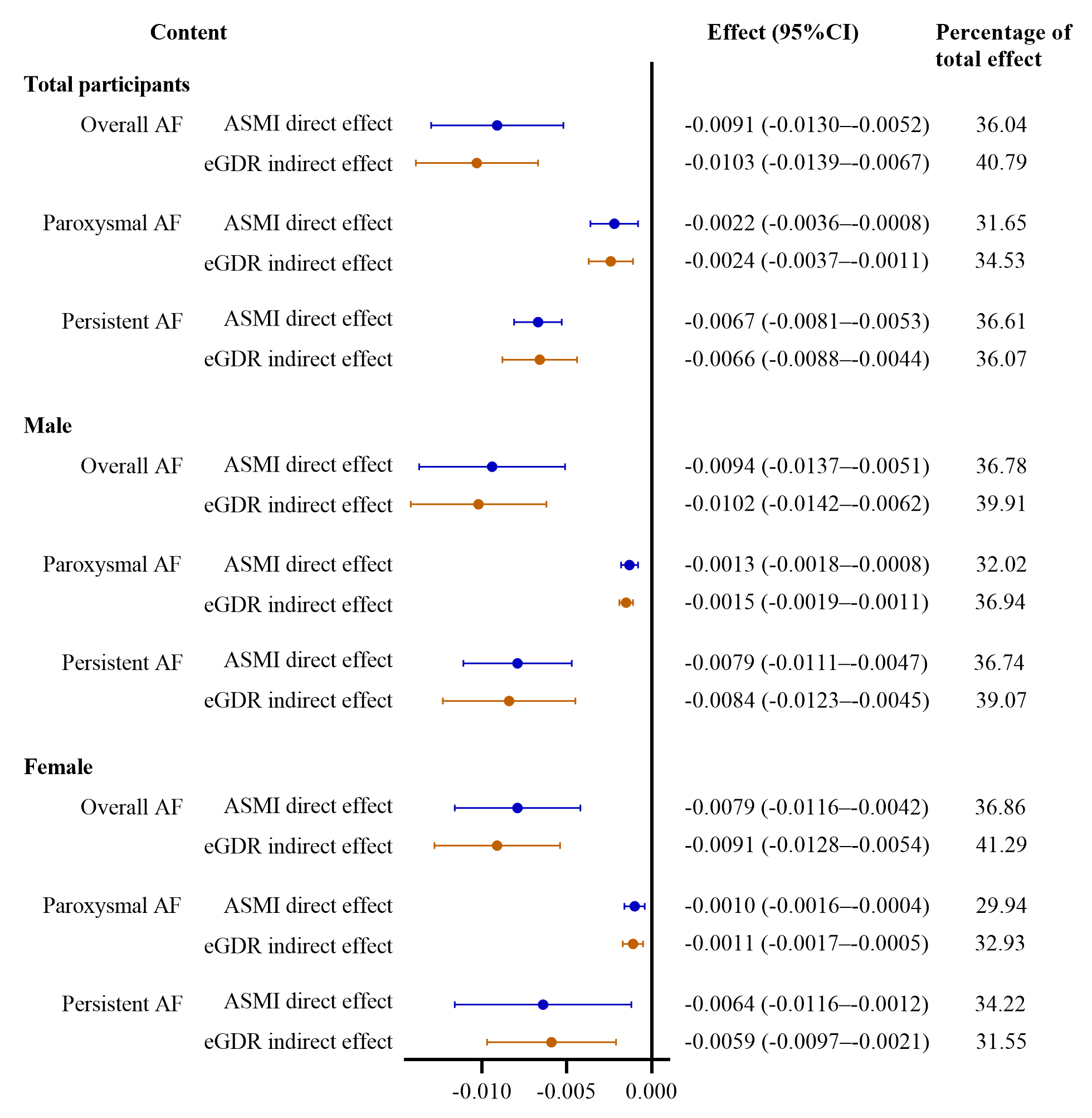
**

**Supplementary Figure 6. Direct effect of ASMI and indirect effect of eGDR on AF risk using mediation analysis.** Models were adjusted for the covariates including age, sex, smoking status, alcohol consumption, exercise, SBP, DBP, heart rate, plasma lipids, FPG, history of dyslipidemia and medications for antihypertension and anti-dyslipidemia, COPD, eGFR, hemoglobin, and CCA-IMT and plaque. ASMI indicates appendicular skeletal muscle mass index; eGDR, and estimated glucose disposal rate.
